# Curcumin: biochemistry, pharmacology, advanced drug delivery systems, and its epigenetic role in combating cancer

**DOI:** 10.3389/fphar.2025.1695200

**Published:** 2025-11-13

**Authors:** Shukur Wasman Smail, Peter Bergsten, Kalthum Othman Taha, Raya Kh. Yashooa, Dawan J. Hawezy, Muhamed Aydin Abbas, Mudhir Sabir Shekha

**Affiliations:** 1 College of Pharmacy, Cihan University-Erbil, Erbil, Iraq; 2 Department of Biology, College of Science, Salahaddin University-Erbil, Erbil, Iraq; 3 Department of Medical Cell Biology, Uppsala University, Uppsala, Sweden; 4 Department of Women’s and Children’s Health, Uppsala University, Uppsala, Sweden; 5 Paediatric Obesity Clinic, Uppsala University Hospital, Uppsala, Sweden; 6 Department of Biology, College of Education for Pure Sciences, University of Al-Hamdaniya, Mosul, Iraq; 7 Department of Surgery, Faculty of General Medicine, Koya University, Koysinjaq, Iraq

**Keywords:** curcumin, epigenetic regulation, RNA methylation, DNA methylation, histone modification, non-coding RNA, drug delivery systems, cancer therapy

## Abstract

Curcumin, the principal bioactive compound of *Curcuma longa* (turmeric), has received extensive scientific attention for its remarkable pharmacological and epigenetic activities, particularly in cancer prevention and therapy. This review provides a comprehensive overview of curcumin’s biochemical, pharmacological, and molecular actions. Curcumin exerts potent antioxidant, anti-inflammatory, and anticancer effects by modulating multiple signaling pathways, including NF-κB, PI3K/Akt, and Wnt/*β*-catenin. Despite its broad therapeutic potential, curcumin’s clinical application is limited by poor solubility, rapid metabolism, and low systemic bioavailability. To address these challenges, advanced nanotechnology-based drug delivery systems such as nanoparticles, liposomes, micelles, and polymeric carriers have been developed to enhance its solubility, stability, and targeted bioavailability. Importantly, curcumin demonstrates a multifaceted epigenetic influence that encompasses the inhibition of DNA methyltransferases leading to DNA demethylation and reactivation of silenced tumor-suppressor genes, modulation of histone acetylation and methylation balance to restore normal chromatin accessibility, regulation of non-coding RNAs such as microRNAs, lncRNAs, and circRNAs that control gene expression, and alteration of RNA methylation (m^6^A modification) through modulation of METTL3, FTO, and YTHDF proteins, which influence mRNA stability and translation efficiency. Collectively, these molecular and epigenetic effects reinforce curcumin’s potential as a promising multi-target agent for cancer prevention and therapy. Further pharmacogenomic and clinical studies are essential to standardize curcumin formulations and translate these preclinical findings into effective therapeutic applications.

## Graphical Abstract

**Figure:**
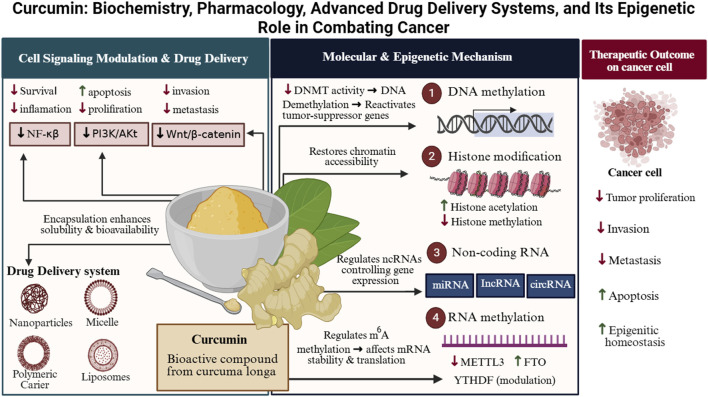

## 1 Introduction

Cancer is the primary cause of mortality in humans and a significant barrier to extending human life expectancy (Chen L. et al., 2021; Zhao et al., 2022). The GLOBOCAN 2020 projection projected 19.3 million new cancer diagnoses and over 10 million cancer-related deaths in 2020 (Sung et al., 2021). Notwithstanding the ongoing progress in biological sciences, cancer incidence and death continue to escalate globally. Numerous factors, such as food, obesity, alcohol dependence, and excessive employment, contribute to the occurrence of various cancers (Raj et al., 2022). Multiple studies have demonstrated curcumin’s therapeutic potential, which includes, but is not limited to, being anticarcinogenic, antioxidant, hypoglycemic, antibacterial, hepato-neuroprotective, and anti-inflammatory, even though no nutritional benefit was discovered (Joe et al., 2004; Hewlings and Kalman, 2017; Heidari et al., 2020).

From a chemical standpoint, curcumin is a hydrophobic polyphenol characterized by low water solubility, low stability, and restricted bioavailability. It may also spontaneously undergo keto-enol tautomerism (Nelson et al., 2017). Despite its potential, curcumin’s clinical applications are limited by poor water solubility, rapid metabolism, and low systemic bioavailability (Anand et al., 2007). Advanced drug delivery systems such as nanoparticles, liposomes, and micelles have been developed to address these challenges. These systems enhance curcumin’s solubility, stability, and targeted delivery, improving therapeutic efficacy while minimizing side effects (Liu et al., 2013). Innovative formulations continue to expand curcumin’s scope in clinical settings, particularly in cancer therapy.

Curcumin is recognized for its significant anticancer properties, which have been investigated through multiple proposed mechanisms of action. Numerous preclinical studies have confirmed these properties, although pharmacokinetic analyses indicate that human plasma concentrations of curcumin are substantially lower than those observed *in vitro* (Cheng et al., 2001; Sharma et al., 2004). Epigenetics refers to the study of heritable changes in gene expression that do not involve alterations to the DNA sequence. Epigenetic modifications regulate essential cellular processes, including proliferation, cell growth, migration, and differentiation, and are characterized as heritable, dynamic, and reversible (Dai et al., 2021). These modifications are closely associated with cancer initiation and progression. Comprehensive analyses of the human cancer genome over the past decade have shown that malignant pathogenesis is partly attributable to epigenetic dysfunction, such as RNA methylation, DNA methylation, non-coding RNA regulation, histone modification, and chromatin structure remodeling (Chen et al., 2022; Gimeno-Valiente et al., 2022).

This review examines the biochemistry, pharmacology, and therapeutic potential of curcumin, with an emphasis on its application in cancer treatment. It discusses challenges such as low bioavailability and highlights advanced drug delivery systems designed to improve efficacy. Furthermore, the review explores curcumin’s roles in epigenetic regulation, linking its molecular characteristics to clinical oncology.

The subsequent sections present a comprehensive overview of the scientific and translational journey of curcumin in cancer research. Section 2 introduces curcumin’s chemical structure and physicochemical characteristics, providing the foundation for understanding its biological behavior. Section 3 addresses the pharmacokinetic challenges associated with curcumin and highlights innovative delivery systems, including nanoparticle, liposomal, and micellar formulations that enhance its bioavailability. Section 4 elucidates the molecular mechanisms underlying curcumin’s anticancer activity, emphasizing its ability to modulate multiple signaling cascades and regulate the epigenetic landscape through DNA methylation, histone modification, and non-coding RNAs. Section 5 synthesizes evidence from preclinical and clinical investigations that collectively demonstrate curcumin’s therapeutic potential and safety profile in oncology. Building upon these findings, Section 6 provides a critical analysis and comparative insight into curcumin’s epigenetic mechanisms, evaluating its specificity, reproducibility, and cross-comparison with other natural epigenetic modulators. Finally, Section 7 explores the unresolved and controversial aspects of curcumin epigenetic research, delineates key limitations, and outlines future directions and translational opportunities for integrating curcumin into evidence-based cancer prevention and therapy.

## 2 Curcumin: biology, chemistry, and mechanism of action

**FIGURE 1:**
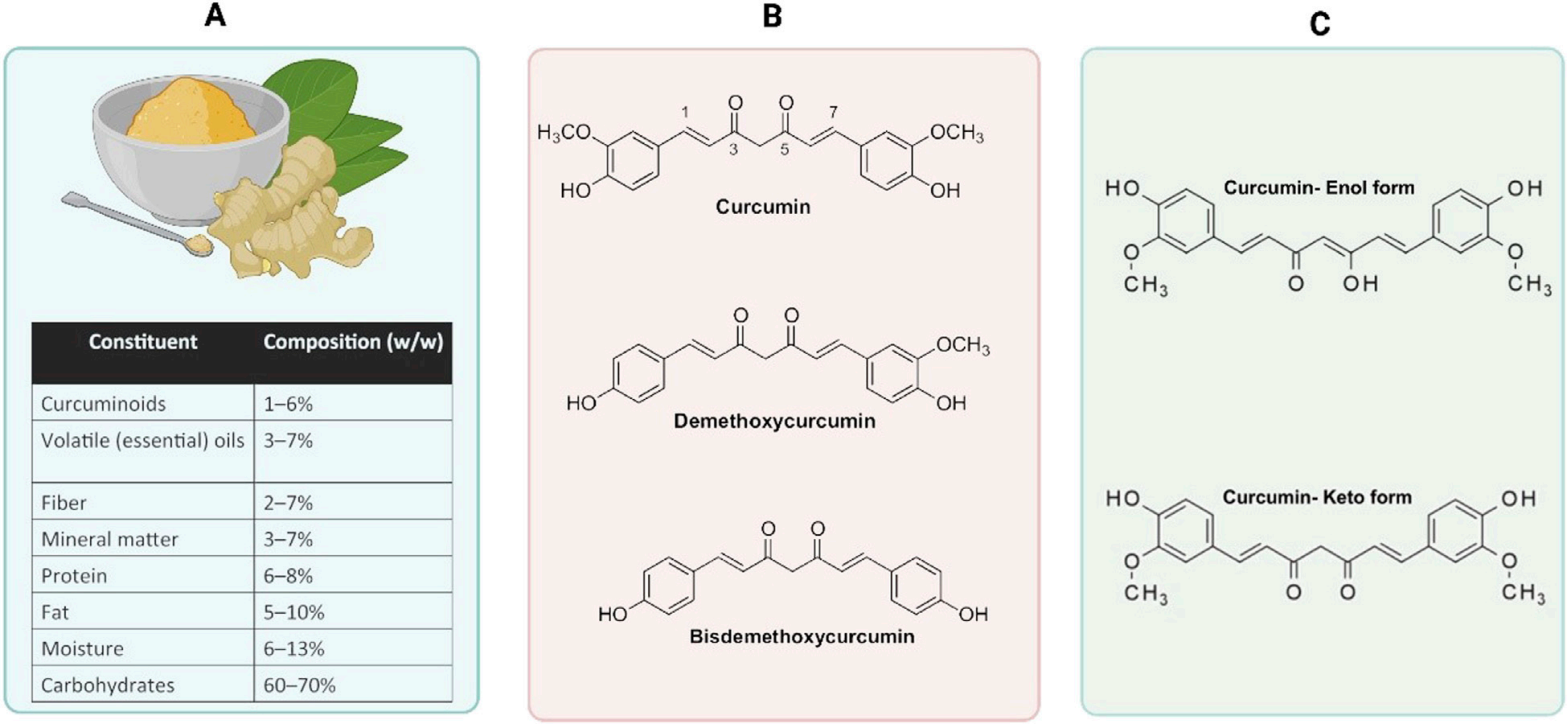
The composition and chemical structure of *Curcuma longa* (turmeric). **(A)** highlights the biological composition of *Curcuma longa*, showing its key constituents with their approximate weight percentages (w/w). These include curcuminoids (1%–6%), volatile essential oils (3%–7%), fiber (2%–7%), mineral matter (3%–7%), protein (6%–8%), fat (5%–10%), moisture (6%–13%), and carbohydrates (60%–70%). A visual representation of turmeric root and powder is also included to depict its natural form. **(B)** illustrates the chemical structures of the primary curcuminoids present in turmeric. These include curcumin, the main bioactive compound with a symmetrical structure comprising two aromatic ring systems substituted with methoxy and hydroxyl groups; DMC, a variant missing one methoxy group; and BDMC, which lacks both methoxy groups. **(C)** focuses on the tautomerism of curcumin, showing its two interconvertible forms. The enol form features a hydroxyl group stabilized across a conjugated system, contributing to its antioxidant properties, while the keto form displays a diketone structure that dominates under certain environmental conditions.

Turmeric powder, derived from the root of Curcuma longa, is widely utilized as both a spice and a traditional medicine in various countries, notably India and China. *Curcuma longa* is a member of the Zingiberaceae family, which also includes ginger (*Zingiber officinale*). The principal phytochemical component of turmeric powder is curcuminoid, comprising 1%–6% of its dry weight. Curcuminoids are composed of three primary constituents: curcumin (60%–70%), demethoxycurcumin (DMC) (20%–27%), and bisdemethoxycurcumin (BDMC) (10%–15%), along with other secondary metabolites of lesser clinical significance (Figure 1) (Priyadarsini, 2014).

Curcumin (diferuloylmethane) (C_21_H_20_O_6_) is chemically [1, (1E,6E)-1,7-bis(4-hydroxy-3-methoxyphenyl)-1,6-heptadiene-3,5-dione (Figure 1). Curcumin, a natural compound found in turmeric, is known for its potential health benefits. However, despite its promising properties, curcumin is chemically unstable (Tønnesen and Karlsen, 1985). This instability is primarily attributed to tautomerization, a process in which curcumin can exist in two forms: enol and keto (Figure 1C). The specific form that curcumin takes is highly dependent on factors such as the solvent used and the pH of the surrounding environment. This tautomeric equilibrium can greatly impact the overall stability and effectiveness of curcumin, making it challenging to use in various applications (Leamy et al., 2011). Researchers are actively exploring strategies to enhance the stability of curcumin and maximize its potential benefits in the fields of medicine, nutrition, and beyond (Kazakova et al., 2022; Kumar A. et al., 2025).

Curcumin is generally recognized as safe by the Food and Drug Administration (FDA) as a food additive, with a recommended daily intake not exceeding 20 mg. Therapeutic applications often require higher doses. Despite extensive research, some scientists classify curcumin as a pan-assay interference compound (PAIN), which challenges its validity as a metabolic panacea (IMPS) or as a suitable lead compound (Nelson et al., 2017). Although curcumin has attracted considerable attention for its potential health benefits and therapeutic properties, certain scientific perspectives question its efficacy and reliability as a drug development candidate. These criticisms stem from concerns about curcumin’s interference with multiple research assays, complicating the assessment of its true biological activity. Furthermore, limited target specificity has been cited as a barrier to its use as a lead compound in drug discovery (El-Saadony et al., 2025). These divergent viewpoints underscore the necessity for further investigation and a comprehensive evaluation of curcumin’s limitations.

Curcumin demonstrates a substantial ability to interact with and modulate the regulation of various proteins, growth factors, transcription factors, enzymes, receptors, and other regulatory genes through both direct and indirect mechanisms (Goel et al., 2008; Chauhan et al., 2025). Extensive research has identified direct targets of curcumin in humans, including protein kinases and reductases, proteasomes, inflammatory molecules, transport proteins, DNMTs, and metal ions (Aggarwal et al., 2007; Sohn et al., 2021). Indirect targets include enzymes, transcription factors, growth factors, receptors, inflammatory mediators, cell cycle regulators, and adhesion molecules (Gupta et al., 2013).

### 2.1 Pharmacokinetics and pharmacodynamics of curcumin

Curcumin has attracted attention in therapeutic areas such as oncology, neurology, and inflammatory diseases. However, its clinical application remains limited due to poor pharmacokinetic and pharmacodynamic properties. Understanding the underlying mechanisms is essential for optimizing curcumin as a therapeutic agent.

#### 2.1.1 Pharmacokinetics of curcumin

Curcumin has low systemic exposure after oral administration due to several pharmacokinetic barriers, including poor aqueous solubility, low absorption, rapid metabolism, and rapid systemic elimination. Upon oral administration, this compound undergoes extensive hepatic and intestinal wall first-pass metabolism to eventually form water-soluble curcumin glucuronide and curcumin sulfate metabolites. These metabolites, though more water-soluble, are less bioactive than the parent compound (Anand et al., 2007).

The lipophilic nature of curcumin significantly limits its absorption through the gastrointestinal tract. Less than 1% of orally administered curcumin is detected in its active form in systemic circulation. Peak plasma concentrations occur within 1–2 h after administration and decline rapidly, with a half-life of approximately 1–2 h. This brief systemic availability necessitates the development of strategies to enhance its pharmacokinetic profile (Shoba et al., 1998; Yadav A. K. et al., 2025).

Several advanced drug delivery systems have been designed to address these limitations. Nanoparticles, liposomes, phytosomes, and micelles have demonstrated efficacy in improving the solubility, stability, and systemic retention of curcumin (Mohanty et al., 2012). Additionally, bioenhancers such as piperine, a component of black pepper, have significantly increased curcumin bioavailability by inhibiting its glucuronidation (Shoba et al., 1998).

##### 2.1.1.1 Metabolism of curcumin

**FIGURE 2:**
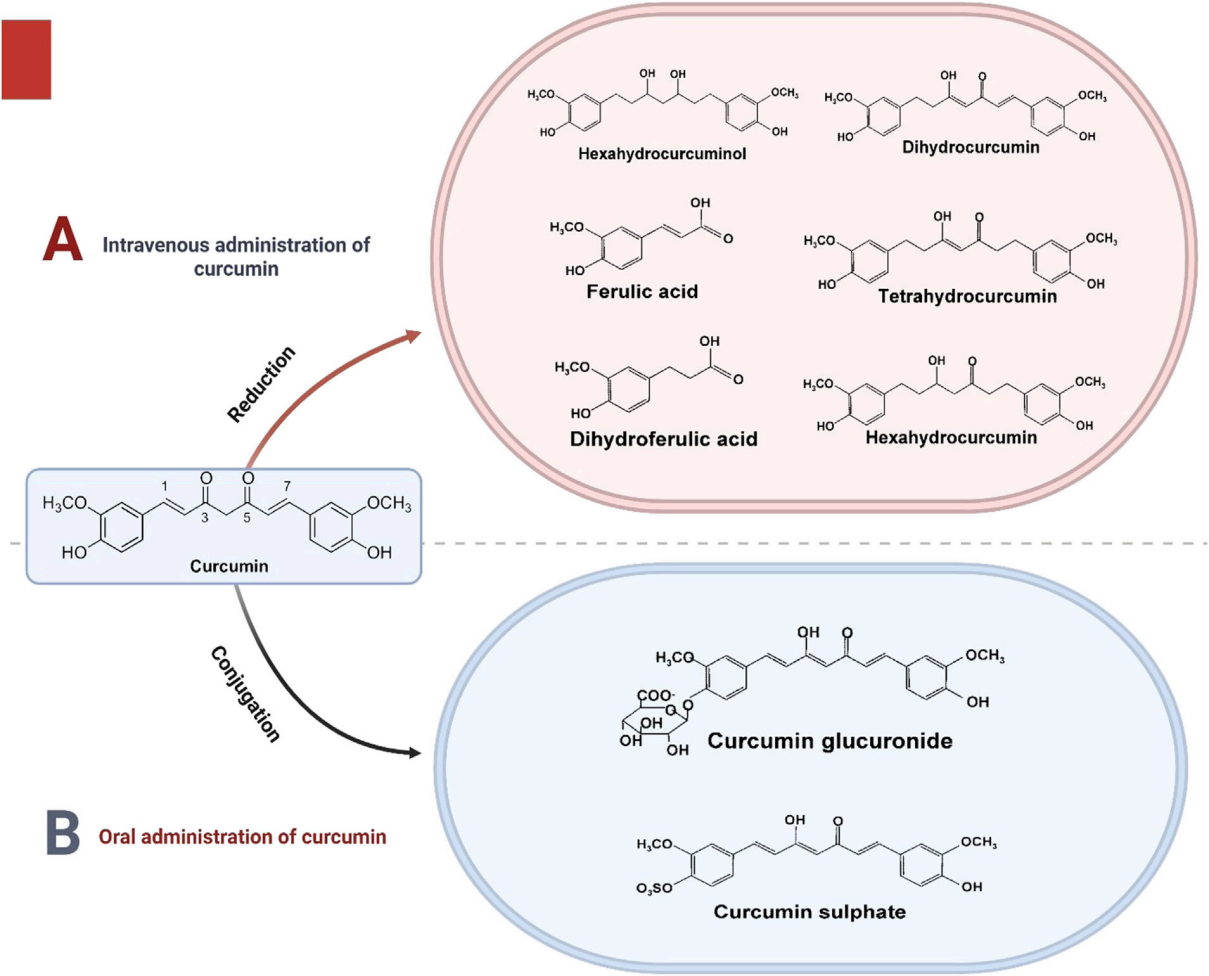
The metabolic pathways and resulting metabolites of curcumin based on its mode of administration. In **(A)**, curcumin administered intravenously undergoes a series of reduction reactions, producing various metabolites such as hexahydrocurcuminol, dihydrocurcumin, tetrahydrocurcumin, hexahydrocurcumin, ferulic acid, and dihydroferulic acid. These reduced metabolites demonstrate the enzymatic transformation of curcumin in the body when introduced directly into the bloodstream. In **(B)**, the figure illustrates the metabolic pathway following oral administration of curcumin. Curcumin undergoes conjugation reactions in the liver, leading to the formation of conjugated metabolites such as curcumin glucuronide and curcumin sulfate. These conjugated forms are the primary metabolites found in circulation after oral consumption, reflecting the role of the digestive and hepatic systems in curcumin metabolism. This figure emphasizes the metabolic differences influenced by the route of administration and highlights the chemical transformations curcumin undergoes in the body.

After being taken orally, curcumin undergoes various metabolic processes in the liver. These include conjugation with sulfation and glucuronidation, resulting in the formation of curcumin sulfate and curcumin glucuronide, respectively (Figure 2). In addition to these metabolites, curcumin also undergoes reduction in the liver when administered intravenously. This reduction leads to the production of several compounds, namely dihydrocurcumin, tetrahydrocurcumin, hexahydrocurcumin, hexahydrocurcuminol, ferulic acid, and dihydroferulic acid (Figure 2). These metabolic transformations play a crucial role in the pharmacokinetics and bioavailability of curcumin, impacting its therapeutic potential and effectiveness (Anand et al., 2007).

##### 2.1.1.2 Bioavailability of curcumin

Two aromatic ring systems in curcumin include o-methoxy phenolic groups connected by a seven-carbon connector made up of an α, β-unsaturated β-diketone moiety (Karthikeyan et al., 2020). Curcumin’s chemical makeup generally renders it less soluble in water at both neutral and acidic pH values. Conversely, curcumin dissolves in ethanol, Dimethyl sulfoxide DMSO, acetic acid, chloroform, acetone, and alkali (Mošovská et al., 2016). Curcumin has a molecular weight of 368.38 g/mol, and its key physicochemical characteristics include lipophilicity (cLogP) and solubility (cLogS). In addition, pharmacokinetic properties such as drug-likeness, gastrointestinal absorption, and blood brain-barrier (BBB) permeability are important to consider when evaluating its ADME profile (Daina et al., 2017).

Curcumin exhibits limited bioavailability due to poor gastrointestinal absorption, extensive first-pass metabolism, and rapid systemic clearance. To address these challenges, strategies such as coadministration with piperine, lipid-based formulations, and nanoparticle microencapsulation have been developed. Piperine has been shown to increase curcumin absorption by up to 2000 percent. These innovations support the therapeutic application of curcumin in clinical and dietary contexts (Shoba et al., 1998; Aggarwal and Harikumar, 2009). Furthermore, recent developments, including self-microemulsifying drug delivery systems (SMEDDS) and natural deep eutectic solvents (NADES), have markedly enhanced curcumin’s solubility and stability. These methods improve curcumin’s efficacy in disease treatment by increasing its systemic bioavailability (Sun et al., 2012; Bashkeran et al., 2024).

#### 2.1.2 Pharmacodynamics of curcumin

Curcumin executes its therapeutic benefits through several molecular mechanisms and has thus shown its pharmacodynamic versatility. This molecule has been considered an antioxidant, anti-inflammatory, and anticancer agent because it acts against key signaling pathways that play important roles in disease development. For instance, curcumin inhibits the nuclear factor-kappa B (NF-κB) pathway primarily by blocking the phosphorylation and subsequent degradation of the inhibitory protein IκBα. This inhibition maintains inhibitor of kappa B IκBα in the cytoplasm, preventing the NF-κB dimer (p50/p65) from translocating to the nucleus and reducing the transcription of NF-κB-dependent pro-inflammatory and survival genes (Singh and Aggarwal, 1995; Jobin et al., 1999; Esmaealzadeh et al., 2024). It modulates the apoptotic pathways by induction of pro-apoptotic proteins like Bax while downregulating anti-apoptotic proteins like B cell lymphoma-2 (Bcl-2) (Bahadar et al., 2024). Curcumin is also known for its antiangiogenic action through its inhibitory activity against vascular endothelial growth factor (VEGF) and matrix metalloproteinases (MMPs), which play a crucial role in tumour progression and metastasis (Kunnumakkara et al., 2017). Furthermore, it suppresses oxidative stress by scavenging free radicals and enhancing the activity of endogenous antioxidant enzymes such as superoxide dismutase (SOD) and catalase (Morya et al., 2025). The pharmacodynamic action of curcumin is dose-dependent, typically requiring higher concentrations to achieve therapeutic efficacy. However, its low systemic bioavailability hampers the attainment of these concentrations without advanced delivery systems or combination therapies.

Various signaling pathways and their components have been identified as potential therapeutic targets for the regulatory effects of curcumin. One objective is the nuclear factor-kappa B (NF-κB) complex, which is crucial to inflammation and immune responses (Shehzad and Lee, 2013). Curcumin blocks IκB kinase inhibitor of κB kinase (IKK) activity, preventing the degradation of IκBα and the translocation of NF-κB to the nucleus, leading to downregulation of NF-κB dependent genes involved in cell growth and survival, such as cyclin D1, COX-2, and Bcl-2 family proteins (Plummer et al., 1999; Sarkar et al., 2009). Curcumin’s inhibition of COX-2 is noteworthy due to its non-selective and non-competitive nature, distinguishing it from specific nonsteroidal anti-inflammatory drugs (NSAIDs). Curcumin does not directly interact with the COX-2 catalytic site; rather, it inhibits COX-2 synthesis indirectly by obstructing transcriptional activation via the NF-κB and AP-1 pathways. Curcumin may also moderately inhibit enzymatic activity at higher dosages, hence enhancing its anti-inflammatory properties (Plummer et al., 1999; Surh et al., 2001). Curcumin possesses antioxidant capabilities by stimulating phase II detoxifying enzymes such as glutathione S-transferase (GST), UDP-glucuronosyltransferase, and heme oxygenase-1 (HO-1). It also enhances the activity of the transcription factor Nrf2, which regulates genes crucial for cellular redox homeostasis and safeguarding cells against oxidative damage (Liu Z. et al., 2016).

Curcumin has been shown to inhibit the phosphorylation of proteins of significant biological importance in various cell lines *in vitro*. For example, in colon cancer cells (PC-3 cell line), at curcumin concentrations ranging from 0 to 50 μM, treatment was associated with decreased phosphorylation of Akt kinase (Akt), mammalian target of rapamycin (mTOR), glycogen synthase kinase (GSK3β), Forkhead box protein O1 (FOXO1), and other proteins (Yu et al., 2008). In breast cancer cell lines, curcumin has been observed to regulate intracellular proteins involved in the Wnt/*β*-catenin signaling pathway at concentrations ranging from 1 to 75 µM. This pathway, known to influence cell proliferation, adhesion, and differentiation, is frequently deregulated in carcinogenesis. Multiple studies indicate that curcumin can negatively regulate cytoplasmic and/or nuclear levels of *β*-catenin in different cell lines (Prasad et al., 2009). Some pharmacodynamic properties of curcumin in various cancers are summarized in Table 1. In humans, curcumin demonstrates measurable pharmacological effects, such as modulation of NF-κB, COX-2, and cytokine signaling; however, its therapeutic application is limited by insufficient bioavailability. Novel formulations are being developed to enhance efficacy and absorption.

**TABLE 1:** The pharmacodynamics changes in various types of cancer by curcumin effect.

Type of cancer	Samples/Models	Dose of curcumin	Observed changes (pharmacodynamics)	References
Breast cancer	MCF-7, MDA-MB-231 (cell lines)	10–50 µM	Inhibition of NF-κB and STAT3 pathways; induction of apoptosis via caspase activation	Aggarwal et al. (2003), Bachmeier et al. (2008)
Colorectal cancer	HCT116, HT-29 (cell lines); xenografts	20–100 µM (*in vitro*); 100 mg/kg (*in vivo*)	Downregulation of COX-2, Wnt/β-catenin pathway suppression; inhibition of tumor growth	Shankar and Srivastava (2007), Patel and Majumdar (2009)
Prostate cancer	LNCaP, PC3 (cell lines)	10–30 µM	Suppression of androgen receptor signaling; inhibition of PI3K/Akt pathway	Dorai et al. (2001), Mukhopadhyay et al. (2001)
Pancreatic cancer	PANC-1, MiaPaCa-2 (cell lines); mouse xenografts	20–40 µM (*in vitro*); 100 mg/kg (*in vivo*)	Inhibition of NF-κB and VEGF; reduced angiogenesis and metastasis	Li et al. (2004), Kunnumakkara et al. (2007)
Lung cancer	A549 (cell line); mouse xenografts	15–30 µM (*in vitro*); 100 mg/kg (*in vivo*)	Induction of apoptosis via mitochondrial pathway; suppression of MAPK and NF-κB pathways	Lin et al. (2009)
Leukemia	HL-60, K562 (cell lines)	5–20 µM	Arrest of cell cycle (G2/M); activation of caspase-3 and PARP cleavage	Khar et al. (1999), Bharti et al. (2003)
Ovarian cancer	SKOV-3 (cell line)	20–40 µM	Downregulation of STAT3 and VEGF; inhibition of proliferation and angiogenesis	Lin et al. (2007)
Glioblastoma	U87, T98G (cell lines)	20–50 µM	Inhibition of JAK/STAT signaling; induction of autophagy and apoptosis	Aoki et al. (2007), Zanotto-Filho et al. (2015)
Colorectal cancer	Phase I clinical trial in advanced colorectal cancer patients	440–2200 mg/day oral curcumin for 29 days	Decreased levels of inducible prostaglandin E2 (PGE2) and COX-2 in colorectal mucosa, indicating anti-inflammatory and anti-proliferative activity	Sharma et al. (2004)
Colorectal cancer (high-risk patients)	Pilot study in patients with aberrant crypt foci	2 g or 4 g/day for 30 days	Reduction in the number of aberrant crypt foci; modulation of M1G (oxidative DNA adducts) levels	Carroll et al. (2011)
Pancreatic cancer	Phase II clinical trial in advanced pancreatic cancer patients	8 g/day oral curcumin	Downregulation of NF-κB, COX-2, and STAT3 expression in peripheral blood mononuclear cells; some patients showed stable disease	Dhillon et al. (2008)
Multiple myeloma	Phase I/II clinical trial	2–8 g/day oral curcumin with/without piperine	Decreased NF-κB activation and IL-6 levels, stabilization of disease in several patients	Jagetia and Aggarwal (2007)
Breast cancer	Randomized controlled trial (RCT) with radiotherapy	Topical curcumin 2% gel	Reduced radiation-induced dermatitis and inflammatory cytokine expression (IL-1, IL-6, TNF-α)	Ryan et al. (2013)

Abbreviations: NF-κB: Nuclear Factor kappa-light-chain-enhancer of activated B cells; STAT3: Signal Transducer and Activator of Transcription 3; COX-2: Cyclooxygenase-2; Wnt/β-catenin: Wingless-related integration site/*β*-catenin signaling pathway; PI3K/Akt: Phosphatidylinositol 3-kinase/Protein kinase B; VEGF: vascular endothelial growth factor; MAPK: Mitogen-Activated Protein Kinase; PARP: Poly (ADP-ribose) Polymerase; JAK/STAT: Janus Kinase/Signal Transducer and Activator of Transcription; PGE2: Prostaglandin E2; M1G: 1,N6-ethenodeoxyguanosine (oxidative DNA, adduct); IL-6: Interleukin-6; TNF-α: Tumor Necrosis Factor-alpha; RCT: randomized controlled trial.

## 3 Epigenetics

Curcumin acts as a potent multi-target epigenetic modulator influencing gene expression through four major mechanisms: it inhibits DNA methyltransferases DNA (Cytosine-5)-Methyltransferase 1 DNA (Cytosine-5)-Methyltransferase 3 Alpha, DNA (Cytosine-5)-Methyltransferase 3 Beta (DNMT1, DNMT3A, DNMT3B), leading to demethylation and reactivation of silenced tumor-suppressor genes; it regulates histone acetylation by suppressing histone acetyltransferases and histone deacetylases, thereby restoring normal chromatin accessibility; it modulates non-coding RNAs by upregulating tumor-suppressive microRNAs (e.g., miR-34a, miR-200c) and downregulating oncogenic ones (e.g., miR-21), influencing proliferation and apoptosis; and, as an emerging mechanism, it affects RNA methylation (m^6^A modification) by altering the activity of key regulators such as METTL3, FTO, and YTHDF proteins, which control mRNA stability and translation. Collectively, these pathways enable curcumin to restore epigenetic homeostasis and counteract cancer progression (Ting et al., 2006).

### 3.1 DNA methylation

Several events have been described by which hypermethylation in CpG islands present within gene promoters contributes to transcriptional silencing. The first is based on the structural change that occurs in methylated cytosines. Because the methyl groups (CH_3_) are projected in the major groove of the DNA, they can block transcription factors, preventing the recognition of the target sequence. A second methylation-mediated mechanism involves methylated DNA-binding proteins (MBPs) (Moore et al., 2013). These molecules interact with methylated DNA, serving as a co-repressor and directly affecting gene silencing. In another context, when abundant CpG sites are present in the gene body, methylation of those nucleotides could lead to gene silencing by blocking transcription. However, intragenic methylation has been associated with increased gene expression (Singal and Ginder, 1999).

**FIGURE 3:**
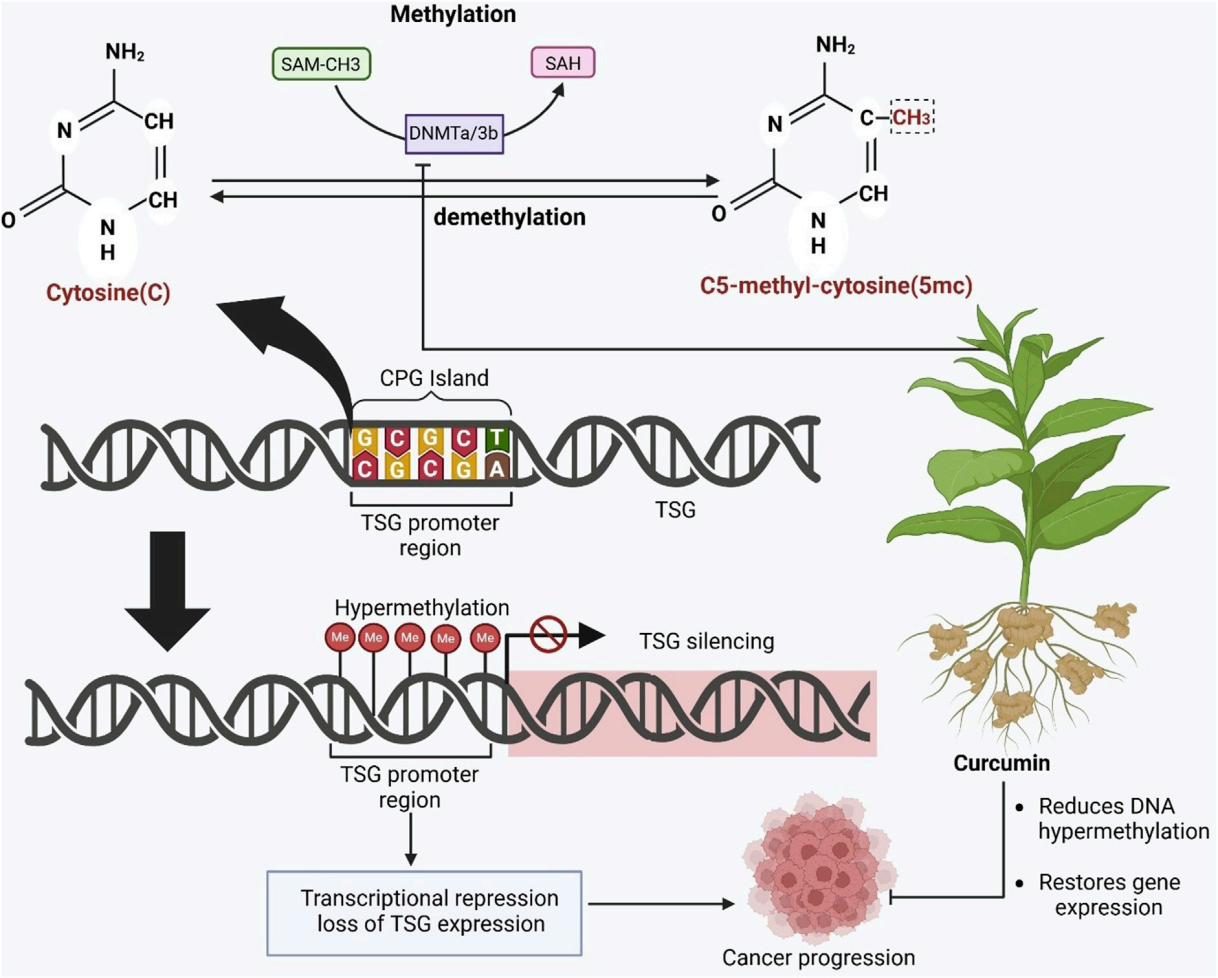
The epigenetic regulation of gene expression through DNA methylation and the role of curcumin in reversing hypermethylation-mediated gene silencing. At the molecular level, cytosine (C) in DNA undergoes methylation at its C5 position to form 5-methylcytosine (5mc), a process catalyzed by DNA methyltransferases (DNMTs), such as DNMTa and DNMT3b, using S-adenosyl methionine (SAM) as a methyl donor and producing S-adenosyl homocysteine (SAH) as a byproduct. This methylation often occurs at CpG islands located in the promoter regions of tumor suppressor genes (TSGs). The hypermethylation of these CpG islands leads to transcriptional repression, silencing the expression of TSGs. This epigenetic silencing prevents the genes from carrying out their tumor-suppressing functions, thereby contributing to cancer progression. The figure depicts this silencing as an accumulation of methyl groups (Me) on the TSG promoter region, leading to transcriptional repression.

DNA methylation and histone modification work together to regulate chromatin structure and gene expression. DNMTs and MBPs recruit chromatin-modifying complexes. This recruitment results in histone deacetylation, which leads to chromatin compaction and gene silencing (Herceg and Ushijima, 2010). DNMTs catalyze the transfer of methyl groups from S-adenosylmethionine to cytosine residues, forming 5-methylcytosine (Figure 3). DNMT3a and DNMT3b act as *de novo* methyltransferases. DNMT1 maintains existing methylation during DNA replication. DNMT2 has a minor maintenance role, and DNMT3L enhances DNMT3a/3b activity (Ting et al., 2006; Yadav S. K. et al., 2025). This methylation system preserves genomic imprinting and tissue-specific gene expression and suppresses oncogene activation during development. In cancer cells, abnormal methylation patterns occur. Promoter hypermethylation of tumor-suppressor genes and global hypomethylation of non-coding regions contribute to chromosomal instability and tumorigenesis (Ting et al., 2006).

#### 3.1.1 Curcumin’s epigenetic modulation: DNA methylation and wnt/*β*-catenin pathway regulation

Curcumin exerts profound epigenetic regulatory effects in cancer by modulating DNA methylation and interfering with the Wnt/*β*-catenin signaling pathway, two mechanisms central to tumorigenesis and cell fate determination.

##### 3.1.1.1 Curcumin as an epigenetic modulator of DNA methylation

Curcumin inhibits DNMTs both directly and indirectly. It suppresses DNMT synthesis and enzymatic activity. This may happen through transcriptional inhibition via NF-κB/AP-1 pathways or by altering methyl donor availability (Hassan et al., 2019). Like 5-aza-2′-deoxycytidine, a known DNMT inhibitor, curcumin downregulates DNMT proteins. This downregulation reactivates silenced tumor suppressor genes such as p16 and RASSF1A (Nagaraju et al., 2013; Shehzad and Lee, 2013; Aggarwal et al., 2015; Pulido-Moran et al., 2016). Studies in colon cancer cell lines (HTC116, HT29, RKO) showed that prolonged curcumin exposure modifies methylation across multiple CpG sites. However, it does not alter global methylation levels as measured by LINE-1 (Link et al., 2013). In neuroblastoma (N2a), curcumin induced NEP promoter demethylation and restored its inhibition of the Akt/NF-κB pathway (Deng et al., 2014). In hepatic stellate cells, curcumin activated PTEN by downregulating DNMT3b and through miR-29b-mediated demethylation (Zheng et al., 2014). Molecular docking analyses revealed that curcumin and its analogs DMC and BDMC form covalent interactions with DNMT1’s catalytic cysteine (C1226). This blocks its thiol (-SH) group and inhibits its activity Experimentally, curcumin treatment reduces DNMT expression levels (Shu et al., 2011; Yu et al., 2013).

Building on these findings, studies show that curcumin causes DNA hypomethylation in leukemia (MV4-11) cells (Liu et al., 2009), It suppresses DLC1 promoter hypermethylation to inhibit breast cancer cell growth (Liu Y. et al., 2017), It also demethylates RARβ in H460 and A549 lung cancer cells (Jiang et al., 2015), and downregulates MAT2A to reduce methylation in hematopoietic stem cells (Lu et al., 2021). Notably, curcumin can enhance methylation at oncogenic promoters like mTOR, increasing DNMT3a/3b expression and suppressing myeloma cell proliferation. Recent research also highlights its effects in lung adenocarcinoma (Kumar M. et al., 2025). Collectively, these studies show curcumin’s bidirectional modulation of DNA methylation and its capacity to sensitize cancer cells to chemotherapeutic agents. Details are in Table 2.

**TABLE 2:** Curcumin effect on DNA methylation on various cancer cells.

Type of cancer	Samples/Models	Dose of curcumin	Changes	References
Lung cancer	H460, A549, and SPC-A-1 cells	0–100 μM	WIF-I promoter hypomethylation and NrF2 promoter demethylation	Liu et al. (2011a)
Colon cancer	AOM- and DSS-enhance colon tumor model (C57BL/6)	Diet consisting of 2% curcumin for a duration of 18 weeks	restores Tnf DNA methylation	Guo et al. (2018)
Gastric cancer	RPMI-1640, MGC-803	0–60 μM	Curcumin decreased the colony formation, proliferation, and migration of cancer cell in a dose- and time-dependent manner. A significant dose of curcumin increased induced mitochondrial damage, ROS levels, apoptosis, and DNA damage in gastric cancer	Tong et al. (2020)
Breast cancer	MCF7 cells	2–20 µM	Declines in all DNMTs	Chatterjee et al. (2019)
Ovarian cancer	ATCC HTB-77 cells	20 μM	Suppresses the expression of the DNMT3a protein and total DNMT activity	Yen et al. (2019)
leukemia	AML cell lines (THP-1, Kasumi-1, and MV4–11)	10 µM or 20 µM	It indicated that curcumin administration induced apoptosis in AML cells and caused them become arrested in the G1 phase of the cell cycle. These processes may co-occur and eventually improve curcumin’s anti-leukemic properties in AML, as demonstrated by our *in vitro* and *in vivo* research	Yu et al. (2013)
Acute lymphoid leukemia	Raji cells	10 μM	Decreases DNMT 1 expression to undo the methylation of the p15 promoter	Sharma et al. (2015)
Prostatic cancer	PC3 and LNCaP cells	14 μM	Suppression of DNMT activity and DNA methylation	Sharma et al. (2016)
Multiple myeloma	NCI-H929 and RPMI-8226 cells	10 μM	Enhances the expression of DNMT3a and DNMT3b to enhance methylation of the mTOR promoter	Chen et al. (2019)
Colorectal cancer	HT29, SW480 and HCT116 cells	2.5μM and 5 μM	Enhances DLEC1 expression and diminishes CpG methylation	Guo et al. (2015)
Hepatocellular carcinoma	Hepa 1–6 cells	1, 5, 10, 25 and 50 μM	Curcumin demonstrated a more pronounced apoptotic effect	Kavoosi (2018)
Pancreatic cancer	MiaPaCa-2 cell lines	10 μM	Emphisze the effect of curcumin to decline methylation	Nagaraju et al. (2013)

Nrf2: nuclear factor erythroid 2–related factor 2, ROS: reactive oxygen species, DNMTs: DNA, methyltransferases,DNMT3a: DNA, methyltransferase 3 alpha, AML: acute myeloid leukemia, DNMT3b: DNA, methyltransferase 3β, mTOR: mammalian target of rapamycin, DLEC1: Deleted in lung and esophageal cancer 1.

##### 3.1.1.2 Curcumin and the Wnt/*β*-catenin signaling pathway

**FIGURE 4:**
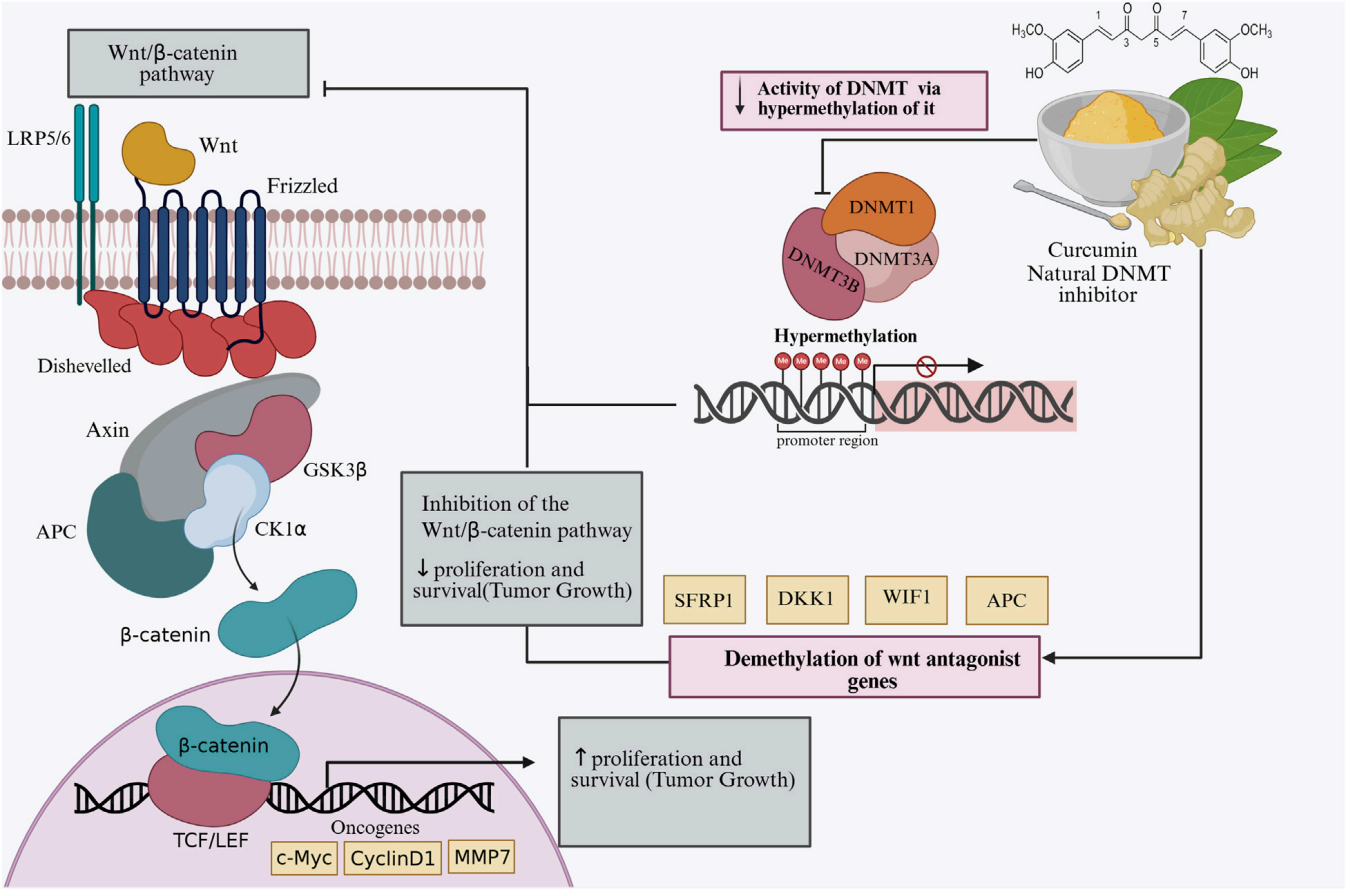
Curcumin-mediated epigenetic regulation of the Wnt/*β*-catenin signaling pathway. Curcumin acts as a natural DNA methyltransferase (DNMT) inhibitor, suppressing DNMT1, DNMT3A, and DNMT3B activity and demthylation of Wnt antagonist genes such as SFRP1, DKK1, WIFI, and APC. This demethylation restores gene expression, leading to inhibition of the Wnt/*β*-catenin pathway, reduced oncogene activation (c-Myc, Cyclin D1, MMP7), and decreased tumor cell proliferation and survival. Abbreviations: DNMT, DNA methyltransferase; SFRP1, secreted frizzled-related protein 1; DKK1, Dickkopf-related protein 1; WIFI, Wnt inhibitory factor 1; APC, adenomatous polyposis coli; GSK3β, glycogen synthase kinase 3 beta; CK1α, casein kinase 1 alpha; TCF/LEF, T-cell factor/lymphoid enhancer factor.

The Wnt/*β*-catenin pathway governs cellular proliferation, adhesion, and differentiation. Its dysregulation is a hallmark of many cancers (Anastas and Moon, 2013). Under physiological conditions, Wnt ligands activate Frizzled (Fz) and LRP5/6 receptors. This leads to Dvl-mediated inactivation of Glycogen Synthase Kinase 3 Beta GSK3β, preventing *β*-catenin degradation. Accumulated *β*-catenin then moves to the nucleus and activates TCF/LEF-dependent transcription (Anastas and Moon, 2013; Daby et al., 2025). Conversely, Wnt antagonists such as dickkopf wnt signaling pathway inhibitor 3 (DKK3), secreted frizzled-related protein 1 (SFRP1), and Wnt Inhibitory Factor 1 (WIF1) inhibit this signaling cascade. They do so by trapping Wnt ligands or blocking receptor interaction (Polakis, 2012), as shown in Figure 4.

The alterations that occur in this signal transduction pathway may have genetic and/or epigenetic origins. One of the frequent epigenetic alterations in hepatocellular carcinoma (HCC) is hypermethylation of the promoters of the Secreted Frizzled-Related Protein (SFRP) gene family, which is a key component in the regulation of the Wnt/*β*-catenin signaling pathway. Similar promoter methylation events have been described in other cancers, including the extracellular regulation genes (WIF1), Dickkopf Wnt Signaling Pathway Inhibitor (DKK1-3), SFRP1, 2, 4, and 5) and intracellular regulatory genes (DACT1-3, Axin, APC). This aberrant methylation pattern has also been observed in E-cadherin and non-transforming Wnt ligands (Wnt5a, 7a, and 9a), leading to constitutive activation of the Wnt/*β*-catenin pathway and uncontrolled cellular proliferation (Ying and Tao, 2009). Curcumin and other compounds [structurally similar to curcumin such as DMC, BDMC, Tetrahydrocurcumin (Portes et al., 2007; Somparn et al., 2007; Srimuangwong et al., 2012; Indira Priyadarsini, 2013)] have been used to re-express abnormally silenced genes by epigenetic mechanisms. Which are involved in the regulation of the Wnt/*β*-catenin signaling pathway (Gao et al., 2009; Liu et al., 2011b).

In non-small cell lung cancer, curcumin and its analogs demethylate the WIF1 promoter, thereby restoring its expression. Specifically, the minimal inhibitory concentrations of DNMT1 activity were reported as 10 μM for curcumin, 5 μM for DMC, and 1 μM for BDMC (Liu et al., 2011a). Following treatment (20 μM, 3 days), significant demethylation and mRNA re-expression of WIF1 were observed, with this effect being especially pronounced with BDMC at lower doses. As a result, the restoration of WIF1 reduced nuclear *β*-catenin levels and consequently suppressed Wnt signaling (Liu et al., 2011b). Importantly, BDMC inhibited DNMT1 activity rather than expression, suggesting direct enzymatic interference.

Overall, the evidence indicates that curcumin acts as a multifaceted epigenetic modulator. It can influence DNA methylation patterns in cancer cells. By inhibiting or downregulating DNMTs (DNMT1, DNMT3A, and DNMT3B), curcumin promotes demethylation of tumor suppressor gene promoters such as p16, RASSF1A, NEP, WIF1, PTEN, and RARβ. This leads to their reactivation and restores normal gene expression. In some contexts, curcumin can also enhance methylation of oncogenic promoters (e.g., mTOR) to suppress tumor growth. These dual regulatory effects suggest that curcumin restores epigenetic balance. As a result, it inhibits cancer cell proliferation, induces apoptosis, and sensitizes tumors to conventional therapies. Consequently, curcumin represents a promising epigenetic therapeutic agent with the potential to reverse aberrant DNA methylation and gene silencing, as illustrated in Figures 3, 4.

### 3.2 Histone modification

**FIGURE 5:**
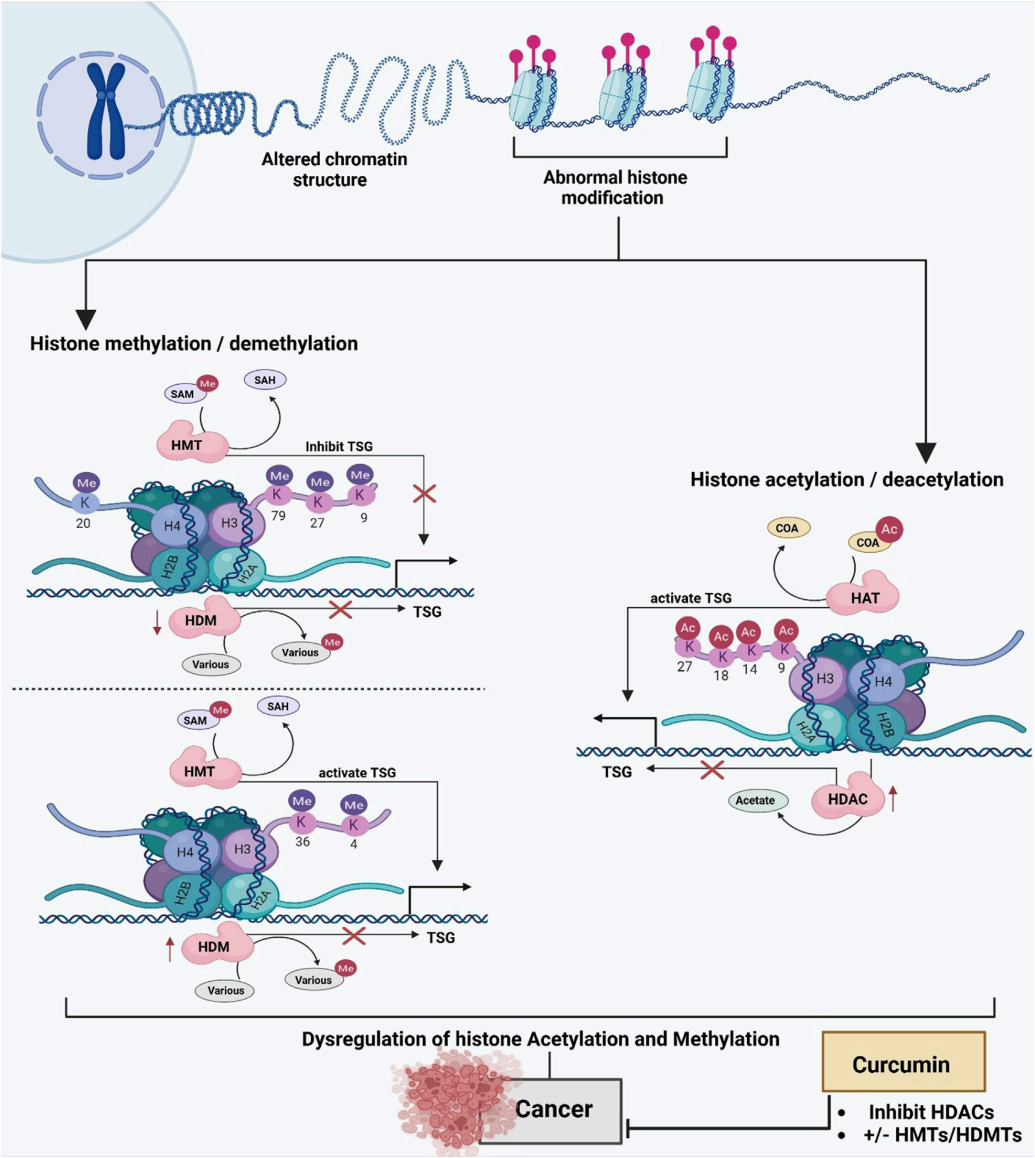
The impact of chromatin structure and histone modifications on gene regulation and how curcumin influences these processes to counteract cancer progression. At the top, altered chromatin structure and abnormal histone modifications are depicted as key contributors to gene silencing, particularly of tumor suppressor genes (TSGs). These modifications include histone methylation/demethylation and histone acetylation/deacetylation, both of which play critical roles in regulating gene expression. On the left, the process of histone methylation and demethylation is shown. Histone methyltransferases (HMTs) add methyl groups (Me) to specific lysine residues on histones (e.g., H3K27, H3K9), using S-adenosyl methionine (SAM) as a methyl donor and producing S-adenosyl homocysteine (SAH). This methylation inhibits TSG expression. Conversely, histone demethylases (HDMs) remove these methyl groups, either repressing or activating TSG expression, depending on the context. On the right, histone acetylation and deacetylation are illustrated. Histone acetyltransferases (HATs) add acetyl groups (Ac) to lysine residues (e.g., H3K18, H4K9), neutralizing the positive charge on histones, relaxing chromatin, and facilitating TSG activation. Conversely, histone deacetylases (HDACs) remove these acetyl groups, leading to chromatin compaction and transcriptional repression of TSGs. At the bottom, the figure highlights the dysregulation of histone acetylation and methylation in cancer, contributing to TSG silencing and tumor progression. Curcumin is shown to counteract these epigenetic changes by inhibiting HDACs and modulating the activity of HMTs and HDMs, thereby restoring TSG expression and reducing cancer progression. This highlights curcumin’s potential as an epigenetic modulator in cancer therapy.

Histone modification is an essential epigenetic event associated with DNA methylation and plays a crucial role in regulating genome integrity, accessibility, and replication. The chromosome of eukaryotes is mainly composed of protein and DNA, and the latter encompasses histone and non-histone proteins. The nucleosome is the fundamental unit of a chromosome, consisting of a core area composed of 146 base pairs of DNA wrapped around a histone octamer, which includes two copies of each of the four core histone proteins: H2A, H2B, H3, and H4. The linker region of the nucleosome comprises a complex of 60 base pairs of DNA and histone H1 (Hao et al., 2021). Histone modification and chromatin remodeling are primarily due to methylation and acetylation (CH_3_-CH_2_) mediated by transferase-type enzymes. In this process, histones H2A, H2B, H3, and H4 can undergo these types of modifications, along with numerous other chemical modifications. The acetylation of lysines or other residues in histones is associated with the activation of transcription, while deacetylation is associated with chromatin compaction and, therefore, with gene silencing (Huang et al., 2014). Histone acetylation and methylation are the most intensively investigated modifications of histone covalent modifications. Histone methylation fundamentally occurs on arginine and lysine residues of the core histone protein via histone methyltransferase (HMT). Additionally, the methyl group can be reversibly isolated by histone demethylase (HDM) (Allis and Jenuwein, 2016; Liu L. et al., 2021). Moreover, histone acetylation is involved in nucleosome assembly, chromatin folding, heterochromatin silencing, and gene transcription. Lysine acetylation residue in the tails of H3 and H4 histone proteins can balance the positive charge of the histone, suppressing the electrostatic interaction and resulting in a decline in the interaction between the DNA backbone and the nucleosome. The transformation between histone deacetylation and acetylation is conserved by histone acetyltransferase (HAT) and Histone deacetylases (HDAC) (Rajan et al., 2020). HAT is manipulated in many processes, consisting of activation of transcription, silencing of genes, cell cycle, and repair of DNA (Figure 5) (Carrozza et al., 2003).

#### 3.2.1 How curcumin regulates histone modification

Curcumin blocks enzymes that add or remove acetyl groups on histones. This changes the balance of histone acetylation. Curcumin can also change the enzymes that add or remove methyl groups, affecting histone methylation. As a result of these changes in histone acetylation and methylation, chromatin accessibility and the transcription of genes regulating cell proliferation and apoptosis are altered. Specifically, H3K9ac is diminished at oncogenic loci, while acetylation is augmented at tumor suppressor loci, contingent upon the context (Dorna et al., 2025; Sadia et al., 2025).

HDAC inhibitors are agents that effectively reduce histone deacetylation; these have been widely used in models in which genes abnormally silenced by epigenetic mechanisms are sought to be re-expressed. However, they are molecules with a high dose-dependent cytotoxicity and associated with the model used. In this context, compounds have become alternatives to the common inhibitors of DNMTs or HDACs. In particular, curcumin has been described as an agent that reduces histone acetylation by inhibiting HAT (histone acetyltransferases), such as the p300/CBP family of proteins (Balasubramanyam et al., 2004; Chen et al., 2007). In the histone modification, curcumin has been detected to be an HDI and has the ability to inhibit the expressions of HDACs, like HDAC1, HDAC3, and HDAC8, with elevated levels of protein of acetylated histone H4 (Liu et al., 2005; Chen et al., 2007; Meja et al., 2008; Hu et al., 2009; Fu and Kurzrock, 2010).

It has been shown that curcumin reduces the global acetylation of histones. Histones, particularly histones H3 and H4, are important in the modulation of gene expression. It has also been described that curcumin, as well as other bioactive phytochemical with similar structures at concentrations between 50 and 500 μM, can specifically inhibit HDACs both at the level of activity and expression. Shu et al. have demonstrated how curcumin could inhibit the total activity of HDACs in prostate cancer cells (LNCaP). Similarly, it was shown that the percentage of H3K27me3 (a modification that promotes transcription repression) in this cell line decreased due to the effect of treatment with curcumin (Shu et al., 2011). The impact of curcumin on these enzymes that modify DNA and histones could depend not only on the concentration of this bioactive phytochemical, it is also important to take into account the cell model used.

Curcumin exhibits varied regulatory implications on histone modifications, including acetylation, methylation, phosphorylation, and glutathionylation across distinct cancer types (Figure 5) (Limberger et al., 2022). In molecular docking models, it has been observed that curcumin fits into the active site of histone deacetylase HDAC8, forming contacts with residues Arg37, Pro35, Ile34, and Phe152 of the catalytic pocket. In addition, this molecule forms two hydrogen bonds, the first between the carboxyl group of Asp29 and the hydroxyl group of curcumin (2.46Å). The second occurs between the carboxyl group of Tyr100 and the oxygen of the phenolic group of curcumin (1.80Å); the binding energy (affinity) between this compound and HDAC was −8.55 kcal/mol (Bora-Tatar et al., 2009). This energy represents the force that is generated in the union between the ligand and the protein. At a general level, it is known that curcumin can inhibit the activity of HDACs 1,3,8,33,36, and HDAC4 (Teiten et al., 2013).

In triple-negative breast cancer, curcumin is noted to reinstate the expression of the tumor suppressor DLC1 by restricting the oncogene Enhancer of zeste homolog 2 (EZH2) through the reduction of Histone 3 lysine 27 trimethylation (H3K27me3) enrichment in the DLC1 promoter, consequently inhibiting the proliferation, migration, and invasion of TNBC cells while achieving apoptosis (Zhou X. et al., 2020). Histone (H3) is the nucleosome protein that involves two residues of cysteine that are able to be changed with S-glutathione. The stabilization of nucleosomes is affected by glutathionylated histone, giving chromatin a loose structure. It is reported that curcumin can moderate histone H3 glutathionylation in MCF7 cells in breast cancer; however, it stimulation the histone H3 acetylation in MCF7 cells (Cianfruglia et al., 2019).

Much evidence suggests that curcumin can inhibit the activity of HDACs at the level of expression and catalytic activity. Lee et al. reported that curcumin induces apoptosis and cell cycle arrest in the G2/M phase in tumor cells, in part by reducing HDAC4 expression and inhibiting its activity (Lee et al., 2011). Other work suggested that curcumin inhibits DNA repair (mediated by Double-Strand Breaks DBS repair) due to its inhibitory effect on HDACs and other effects reported by Wang et al. This effect is beneficial in cancer because chemotherapy and radiotherapy cause DNA damage; in this context, preventing the activation of DNA repair pathways in cancer cells would contribute to a therapeutic effect (Lee et al., 2011).

Given its activity against HDACs, curcumin has multiple anticancer effects. This effect is also partially enhanced by its duality as an inhibitor of HATs (Balasubramanyam et al., 2004; Chen et al., 2007). On the other hand, its HAT inhibitory activity may differ from what was observed on HDAcs and cause different effects in other models. It may also be associated with adverse effects during development (Xia et al., 2012).

In general, many of the anticancer effects of curcumin are related to the simultaneous modulation of several epigenetic mechanisms, with studies suggesting the negative regulation or inhibition of DNMTs and HDACs jointly by this polyphenol (Shu et al., 2011). Even curcumin may have a synergistic effect with HDAC inhibitors in the regulation of signaling pathways. In a recent study, the administration of HDAC inhibitors in combination with curcumin enhances the impact of the phytoconstituent compound. In this same work, it is described that curcumin could inhibit HDACs through a mechanism related to the inhibition of the NF-kB pathway in “cancer stem cells” (Marquardt et al., 2015). The previous study has reported that curcumin enhances the suppression of HDAC activity and inhibits HDAC8 activity of isoform, elevating the suppressor’s cytokine signaling expression (SOCS1, SOCS3) in the leukemic cell (Chen et al., 2013).

A more recent study confirms the inhibitory capacity of curcumin on HDACs. Omotuyi et al. reported that curcumin affects the functioning of the ε-N-acetyl-lysine binding pocket of HDAC1, interfering with its functioning system (charge relay system). In addition, curcumin binds to the catalytic pocket and induces pocket compaction. Taken together, this evidence supports the antitumor activity of curcumin in relation to its ability to modulate DNMTs and HDACs (Omotuyi et al., 2015). The effects of curcumin on histone modifications are detailed in Table 3.

**TABLE 3:** The effects of curcumin on histone modification in various cancer cells.

Type of cancer	Histone modification	Effects	Models	References
Triple-negative breast cancer	Histone methylation	Suppress the expression of EZH2	MDA-MB-231 xenograft tumor	Zhou et al. (2020b)
Prostate cancer	Histone methylation	Suppresses the pathway of JNK and inhibites H3K4me3	LNCaP cells and xenograft tumor	Zhao et al. (2018)
Hepatocellular carcinoma	Histone methylation	Reduces EZH2 to downregulate signaling Wnt/3-catenin	Hep3B and SMMC-7721 cells and xenograft tumor	Khan et al. (2021)
Lung cancer	Histone deacetylases	CU17 enhanced the antiproliferative efficacy of Gem in A549 cells, suggesting its potential as an adjunct therapy to augment the chemotherapy impact of Gem in lung cancer	A549 cells	Namwan et al. (2022)
Hepatocellular carcinoma	Histone acetylation	Upregulates the expression Erα	Hepa 1–6 cells	Sanaei et al. (2020)
Prostate cancer	Histone acetylation	Upregulates the expression of p53 and activates the acetylation of H3 and H4.	LNCaP, DU145, and PC3 cells	Shankar and Srivastava (2007)
Breast Cancer	Histone acetylation	Elevates histone acetylation	MCF7 and MDA-MB-231 cells	Mukherjee et al. (2012)
Bladder cancer	Histone acetylation	Raises acetylation of H3 and H4	RT112, UMUC3 and TCCSUP cells	Roos et al. (2019)
Leukemia	Histone acetylation	Elevates acetylation of H3 and H4	HL-60 cells	Chen et al. (2010)
Colorectal cancer	Histone acetylation	promotes ubiquitination-mediated degradation of the SIRT1 protein	HCT-116, HCT-15, DLD-1 cells	Lee et al. (2018)
Acute leukemia	Histone acetylation	Decline expression of HDAC1, HDAC3, p300	Raji cells	Chen et al. (2007)
Lung cancer	Histone phosphorylation	Elevates phosphorylation of p53 and histone H2A.X	NCI-H460 cells	Ting et al. (2015)
Medulloblastoma	Histone acetylation	Rstracted HDAC activity	DAOY, D283 Med and D341 Med cells	Lee et al. (2011)
Breast Cancer	Histone acetylation	Causes the H3 histone to become acetylated and less glutathionylated	MCF7 cells	Cianfruglia et al. (2019)

EZH2: Enhancer of zeste homolog 2, JNK: Jun N-terminal kinase, H3K4me3: Trimethylated H3 lysine 4, CU17: curcumin derivative, SIRT1: Sirtuin 1, HDAC1,3: histone deacetylase, H2A.X: histone family member X, HDAC: histone deacetylases.

### 3.3 Non coding RNA (microRNA, lncRNAs, and circRNAs)

Non-coding RNAs including miRNAs, long non-coding RNAs (lncRNAs), and circular RNAs (circRNAs), play crucial roles in the post-transcriptional regulation of gene expression, epigenetic control, and cellular signaling pathways in both physiological and pathological conditions. Dysregulation of these ncRNAs has been closely linked to tumor initiation, progression, and chemoresistance, making them promising biomarkers and therapeutic targets in cancer biology (Hombach and Kretz, 2016).

**FIGURE 6:**
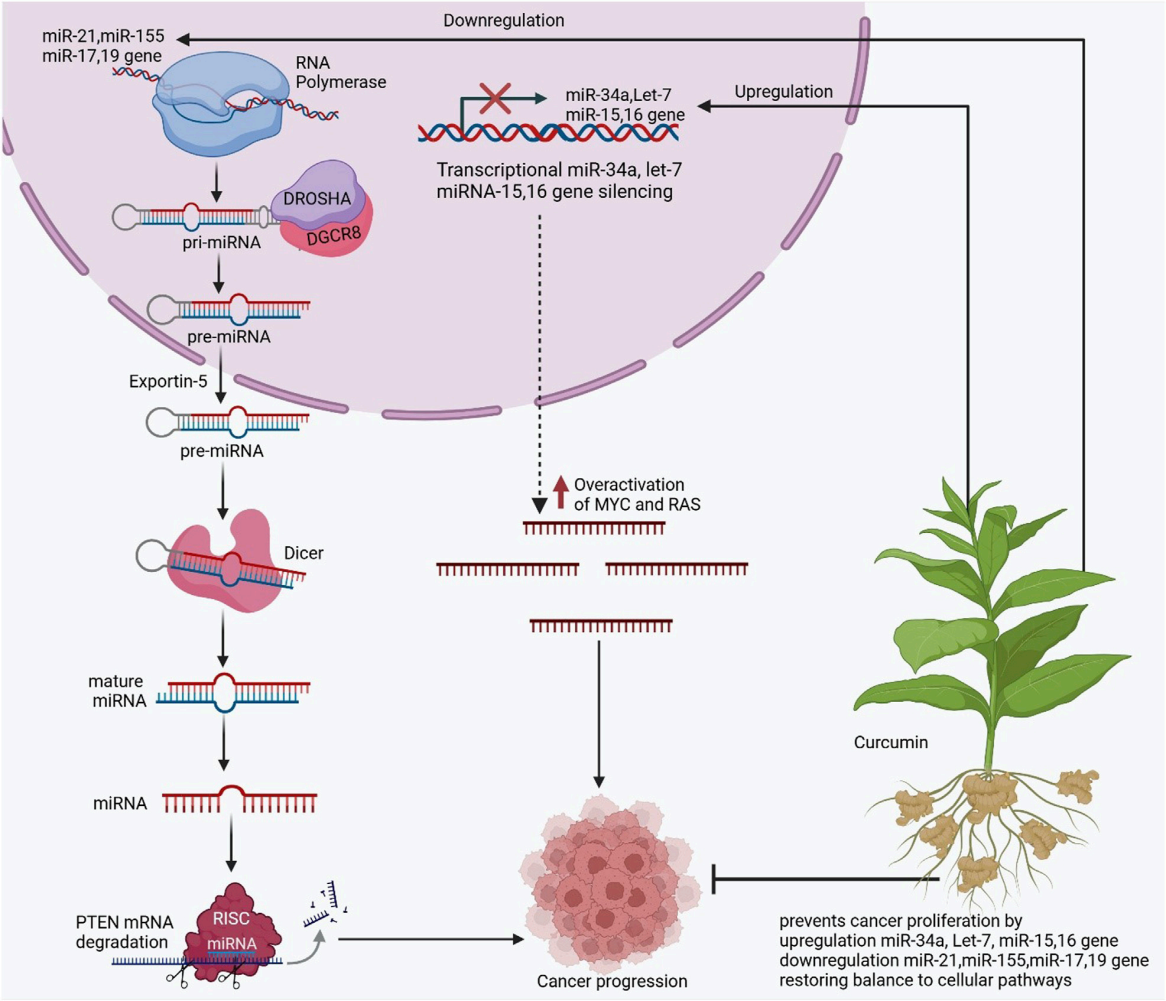
The regulation of microRNAs (miRNAs) in cancer progression and the therapeutic role of curcumin in restoring miRNA balance. miRNAs are small non-coding RNA molecules that regulate gene expression post-transcriptionally. Their biogenesis begins with RNA polymerase-mediated transcription of primary miRNA (pri-miRNA), which is processed by the DROSHA-DGCR8 complex into precursor miRNA (pre-miRNA). Exportin-5 transports the pre-miRNA into the cytoplasm, where it is cleaved by Dicer to form mature miRNA. The mature miRNA associates with the RNA-induced silencing complex (RISC) to target mRNA, such as PTEN, for degradation or translational repression. The figure highlights dysregulation in miRNA expression associated with cancer. Overexpression of oncogenic miRNAs (e.g., miR-21, miR-155, miR-17, and miR-19) leads to degradation of tumor suppressor mRNAs, promoting cancer progression. Concurrently, downregulation of tumor-suppressor miRNAs (e.g., miR-34a, let-7, miR-15, and miR-16) results in overactivation of oncogenes such as MYC and RAS, further driving malignancy. Curcumin is depicted as a modulator of miRNA expression, restoring balance to cellular pathways. It prevents cancer proliferation by upregulating tumor-suppressor miRNAs (e.g., miR-34a, let-7, miR-15, and miR-16) and downregulating oncogenic miRNAs (e.g., miR-21, miR-155, miR-17, and miR-19).

microRNA (miRMA) is 18–25 nucleotides, small, non-coding RNAs that regulate gene expression at the post-transcriptional level and are involved in RNA interference RNAi via connecting to the untranslated region (UTR) of messenger RNA (mRNA) to inhibit translation of protein or mRNA decay (Figure 6) (Ørom et al., 2008). Previous studies have reported that each miRNA targets multiple mRNAs (Dostie et al., 2003). Over 30% of human genes are believed to have been targeted by miRNAs, indicating that miRNAs exert a widespread influence on the transcriptomes and proteomes of eukaryotes (Jaenisch and Bird, 2003; Alarcón et al., 2015). The evidence showed that miRNAs play an essential role in many biological processes involving differentiation, cell proliferation, hematopoiesis, and apoptosis (Dostie et al., 2003; Xu et al., 2003; Chen et al., 2004; Wang et al., 2009). The expression levels of miRNAs can serve as indicators for many disorders. Aberrant miRNAs are recognized as a form of epigenetic modification. There is a growing body of research investigating the mechanisms underlying the dysregulation of miRNAs. A prior study has demonstrated that miRNA expression is regulated by epigenetic mechanisms, such as DNA methylation, RNA modification, and post-translational modifications of histones (Seven et al., 2014). The expression of miRNAs changes epigenetically in both pathological and physiological conditions in a specific tissue manner and is very dynamic and complex; the evidence elevated the expression of miRNA modification among disease state and healthy, indicating the methylation of disease-specific patterns (Van den Hove et al., 2014). In addition, epigenetic monitoring of miRNA can be a miRNA-specific and epigenetic-specific effector (Fabbri et al., 2013).

#### 3.3.1 Curcumin’s function in microRNA control

Curcumin influences the transcription and stability of miRNAs by upregulating tumor-suppressive miRNAs and downregulating oncomiRs, alters the expression of lncRNAs such as Metastasis-Associated Lung Adenocarcinoma Transcript 1 (MALAT1) and Urothelial Carcinoma-Associated 1(UCA1), and modifies the networks of circRNAs, specifically the miRNA sponging axes. Consequently, post-transcriptional regulation of several downstream pathways (Wnt, mTOR, EMT regulators) influences cellular growth, dissemination, and drug resistance (Liu et al., 2019; Wang et al., 2021).

Previous studies indicated that miRNAs are essential targets for curcumin, and curcumin mediates its curative effects (Mirzaei et al., 2016b; Momtazi et al., 2016). Another epigenetic mechanism regulated by curcumin is the production of small non-coding RNAs or miRNAs, which can increase or decrease the expression of a gene. Many reports show that cells treated with curcumin respond by overexpressing and downregulating different miRNAs (Reuter et al., 2011). Among these RNAs, curcumin has been observed to increase the expression of miRNA-16, miRNA-143, miRNA-3436 and miRNA-22,33 within the most outstanding; for example, miRNA-34 is an important cell cycle regulator, contributing to the inhibition of invasion and migration. It has also been reported that this polyphenol positively regulates miRNA-15 and 16, which results in the induction of apoptosis in breast cancer cells (Chang and Yu, 2016). Studies detected that dietary factors such as curcumin have appropriate anti-cancer properties (Mirzaei et al., 2016a); moreover, the exerted agents are influenced by a multiple of molecular and cellular pathways such as Mitogen-activated protein kinases (MAPK), Phosphatase and Tensin Homolog deleted on chromosome 10 (PTEN), signal transducer and activator of transcription (STAT), and a network of miRNA (Figure 6). The use of those agents is associated with many advantages, such as anti-toxic properties (Mirzaei et al., 2017).

Progression of the cell cycle and proliferation sustained are two hallmarks of cancer that have an essential role in normal cells transforming into cancerous ones and the progression of the tumor (Hanahan and Weinberg, 2000). Elevating the levels of reactive oxygen species (ROS) in colon adenocarcinoma cells is one of the outcomes of treatment with curcumin, which results in a decline in cell proliferation and viability (Agarwal et al., 2018). In paclitaxel-resistant non–small cell lung cancer (NSCLC) cells, curcumin enhances sensitivity to paclitaxel by inducing S-phase cell cycle arrest and reducing metastasis-associated protein 1 (MTA1) expression through miRNA-30c–mediated regulation (Lu et al., 2017). Curcumin has demonstrated efficacy in inhibiting the proliferation of bladder cancer cells by targeting the tumor surface antigen such as trophoblast antigen 2 (Trop2), a member of the calcium signal transducer gene family (Zhang et al., 2015). It has an essential role in targeting various pathways like phosphoinositide-3-kinase–protein kinase B/Akt (PI3K/AK), which is another pathway of curcumin to implement its influence on cancer cells, thus suppressing proliferation (Sadoughi et al., 2022).

The studies emphasized that miR-21 is one of the most considerable miRNAs; the over-expression of miR-21 belongs to multiple forms of proliferation of various types of cancer cells (Bautista-Sánchez et al., 2020). Curcumin has the binary influence of miR-21 on colorectal cancer cells via declining the expression, meanwhile restricting the linking of AP-1 to the miR-21 gene promoter and elevating the mir-21 target gene Programmed Cell Death 4 (pdcd4). *In vitro* G2/M arrest and declined proliferation (Mudduluru et al., 2011). In colorectal cancer, miR-21 is the target phosphatase, and PTEN is a protein which is overexpressed via curcumin, while in non-small cell lung cancer is under-expressed (Zhang et al., 2010). However, the miR-21 expression by the influence of curcumin is downregulated in hepatocellular cancer cells, which stimulates the expression of metalloproteinase-3 (TIMP3) and suppresses the GF-b1/smad3 signalling, resulting in the proliferating of cancer cells (Li et al., 2020). Moreover, SRY-Box Transcription Factor 6 (SOX6) levels are elevated following the inhibition of miR-21-5p by curcumin, providing an additional rationale for the reduced proliferation observed in these cells (Zhou C. et al., 2020). Curcumin has been found to modify the expression of miR-192-5p in lung cancer cells. A study demonstrated that curcumin upregulates this miRNA, resulting in the suppression of PI3K/Akt signalling and a subsequent reduction in the proliferation rate of A549 cells (Jin et al., 2015).

Other studies detected the impact of curcumin on miRNAs in metastasis and angiogenesis. Research indicates that curcumin influences the metastasis of certain malignancies by targeting metastasis (Talmadge and Fidler, 2010; Sadoughi et al., 2022). The enhanced production of growth hormone (GH) induces epithelial-mesenchymal transition (EMT) through miR-182-96-183 in breast cancer cells. Administering curcumin to cells for 48 h inhibits the autocrine growth hormone-mediated activation of miR-182-96-183. Consequently, curcumin inhibits invasion, epithelial-mesenchymal transition activation, and metastasis via reducing NF-κB signalling and the clustered expression of miR-182-96-183 (Coker-Gurkan et al., 2019). Curcumin regulates the expression of miR-181b in metastatic breast cancer cells. Consequently, miR-181b diminishes CXC motif chemokine ligand 1,2 (CXCL1) and (CXCL-2) via interacting with their 3′ untranslated region (3′ UTR). Furthermore, the overexpression of miR-181b inhibits the development of metastasis *in vivo* (Kronski et al., 2014). Elevated levels of miR-125a-5p are associated with enhanced migration, proliferation, and invasion. Curcumin treatment of nasopharyngeal cancer cells has demonstrated downregulation of hsa-miR-125a-5p, has-miR-210, and hsa-miR-547-3p expression. Curcumin also enhances TP53 expression, which is inhibited by miR-125a-5p (Gao et al., 2014).

Several investigations have demonstrated that curcumin influences the apoptosis of various cancer types via targeting miRNAs. It was revealed that curcumin induces considerable and dose-dependent inhibition of miR-21 in non-small cell lung cancer cells. Furthermore, curcumin-treated A549 cells exhibited elevated levels of PTEN, a target of miR-21, as demonstrated by Western blot examination. While these pathways lead to curcumin-induced apoptosis, the transfection of cancer cells with miR-21 mimic or PTEN small interfering RNA (siRNA) negates the anticancer properties of curcumin (Zhang et al., 2014). Curcumin regulates the expression of miR-181b in metastatic breast cancer cells. Consequently, miR-181b diminishes CXCL1 and CXCL-2 by interacting with their 3′ UTR. Furthermore, the overexpression of miR-181b inhibits the development of metastasis *in vivo* (Kronski et al., 2014). Curcumin therapy markedly elevates miR-9 expression in SKOV3 ovarian cancer cells, triggering apoptosis. Overexpression of MiR-9 enhances caspase-3 degradation and poly (ADP-ribose) polymerase activity. Moreover, curcumin-induced overexpression of miR-9 leads to the subsequent alteration of the Akt/forkhead box protein O1 (FOXO1) pathway (Zhao et al., 2014). Chen et al. (2020) demonstrated that curcumin exerts its pro-apoptotic effect, at least in part, by inhibiting miR-21. They additionally discovered that curcumin demonstrates anticancer benefits against large B-cell lymphoma that is diffuse via Von Hippel-Lindau (VHL), which is a direct target of miR-21 (Chen et al., 2020). Tan et al. (2018) revealed that the combination of an antisense oligonucleotide targeting miR-21 and curcumin is more effective in boosting PDCD4, PTEN, and apoptosis in glioblastoma cells than curcumin alone. In bladder cancer, curcumin induces the downregulation of miR-7641, a tumor-promoting miRNA. Consequently, p16, a target of miR-7641, is elevated, resulting in accelerated apoptosis (Wang K. et al., 2018). Saini et al. (2011) also demonstrated that curcumin promotes miR-203, which is regarded as a tumour suppressor miRNA, in bladder cancer. Curcumin induces hypo-methylation of the miR-203 promoter and enhances miR-203 expression. Consequently, the target genes of miR-203 (Akt2 and Src) are downregulated, triggering apoptosis (Saini et al., 2011). Curcumin causes apoptosis in two retinoblastoma cell lines, SO-Rb50 and Y79. Cells treated with curcumin have elevated levels of miR-99a. Furthermore, curcumin suppresses the phosphorylation of Janus kinase 1 JAK1, STAT1, and STAT3. However, berberine does not exhibit any inhibitory effect on the JAK/STAT signalling pathway in miR-99a knockdown cells (Li et al., 2018).

As previously said, in addition to the negative effects of chemotherapy, the resilience of cancer cells is another factor that has driven us to explore alternative therapeutic options. It may also be feasible to eliminate this hurdle by reducing the resistance (Sadoughi et al., 2022). Lung cancer, in particular, has consistently had the greatest incidence of newly diagnosed patients compared to other malignancies for years and is typically treated with paclitaxel-based chemotherapeutic agents (Park et al., 2016; Schabath and Cote, 2019). Resistance to paclitaxel-based therapies is the primary reason this malignancy ranks as the leading cause of cancer-related mortality in men and the second highest cause in women (Park et al., 2016; Schabath and Cote, 2019). In a study conducted via Lu et al. (2017), the application of curcumin affects the levels of miR-30c and, therefore, MTA1. Upregulating miR-30c to diminish the expression of this gene enhances the sensitivity of aggressive NSCLC to paclitaxel treatment (Lu et al., 2017).

A cohort of researchers investigated a curcumin analogue, cation diffusion facilitator (CDF), on gemcitabine-resistant pancreatic cancer cells to target cancer stem cells. They determined that the downregulation of signalling pathways in cancer stem cells is associated with miR-200 and miR-21. In gemcitabine-resistant cells, miR-200 and miR-21 exhibit elevated and diminished levels, respectively; however, CDF can reverse this expression profile and enhance treatment effects (Ali et al., 2010). Monteleone et al. (2018) observed that curcumin enhanced the antileukemic effect of imatinib in chronic myelogenous leukemia (CML) cells *in vitro* by modulating the miR-22/IPO7/HIF-1α axis and altering the metabolic enzyme profile (Monteleone et al., 2018). In glioblastoma cells, Wu et al. (2015) showed that chemosensitization to temozolomide can be achieved by the upregulation of miR-146a and the inactivation of NF-κB resulting from curcumin treatment. Apoptosis is triggered in U-87MG cells by the combination of curcumin and temozolomide (Wu et al., 2015). Zhou et al. (2017) investigated the utilization of miRNA signalling pathways influenced by curcumin to reduce the resistance of breast cancer cells to Adriamycin. MiR-29b-1-5p mediates the anti-resistance effects of liposomal curcumin on MCF-7 cell line (Zhou et al., 2017). The effects of curcumin on miRNA are detailed in Table 4.

**TABLE 4:** The curcumin effect on miRNAs regulation.

Type of cancer	ncRNAs regulation	Target	Biological function	References
Lung cancer	↑miR-98	↓LIN-28A	Suppress cell Migration and invasion	Liu et al. (2017b)
Bladder cancer	↓miR-7641	↑p16	Decline invasion and elevate apoptosis of cancer cells in bladder	Wang et al. (2018a)
Ovarian Cancer	↑miR-9	↓Akt, FOXO1	Cytotoxic effects on SKOV3 ovarian cancer cells primarily via overexpression of miR-9 and consequent alteration of the Akt/FOXO1 axis	Zhao et al. (2014)
Breast cancer	↓miR-21	↑PTEN/Akt	showed the anticancer impact of curcumin in reducing breast cancer cell proliferation, and has explained that the miR-21/PTEN/Akt signaling pathway is a critical mechanism for the anticancer actions of curcumin	Wang et al. (2017)
esophageal squamous cell carcinoma	↑miR-532-3p	↓AKT	Curcumin suppressed AKT phosphorylation by enhancing miR-532-3p expression, thereby decreasing the activation of the AKT pathway. In conclusion, curcumin serves as a strong inhibitor of ESCC proliferation, mediated through the modulation of the circNRIP1/miR-532-3p/AKT pathway	Luo et al. (2023)
Breast cancer	↑miR-15a-5p	↓CCNE1 and BMI1	Inhibit the cell viability in breast cancer cell	Suer et al. (2024)
Ovarian cancer	↓miR-133b	↓GSTP-1	the possibility of curcumin to augment the efficacy of cisplatin in the treatment of ovarian cancer. Participation in resistance to drugs and underscores their value as targets for therapy	Ülker et al. (2024)
Glioblastoma cells	↓miRNA-21	NA	Curcumin plays a crucial impact in downregulating miRNA-21 expression and activating extrinsic and intrinsic apoptotic pathways in LN-18 cells	Razali et al. (2024)
Colorectal cancer	↓miR-130a	↑Nkd2	Suppresses the proliferation of cell	Dou et al. (2017)
Prostatic cancer	↑miR-145 ↓lncRNA-ROR	↑OCT4	Cell cycle arrests	Liu et al. (2017a)
Leukemia	↑miR-20a-5p, ↓lncRNA-HOTAIR	↓WT1	Prohibits the proliferation of cell and movement, arrests cell cycle.	Liu et al. (2021a)
Gastric cancer	↓lncRNA-H19	↑p53	Inhibits the proliferation of cell in addition to enhances apoptosis	Liu et al. (2016a)
Non-small cell lung cancer	↑miR-206	↓Phosphorylation mTOR level and AKT	Suppresses migration of cell and invasion	Wang et al. (2020)

LIN-28A: Lin-28, Homolog A, p16: tumour suppressor gene, ESCC: esophageal squamous cell carcinoma, CCNE1: Cyclin E1, BMI1: B lymphoma Mo-MLV, insertion region 1 homolog), GSTP-1: Glutathione S-transferases P1, Nkd2: naked endosperm 2, OCT4: octamer binding transcription factor 4, WT1: Wilms’ tumour gene 1.

#### 3.3.2 Curcumin’s function in lncRNAs and circRNAs control

Curcumin is a strong regulator of other non-coding RNAs, like lncRNAs and circRNAs, in addition to changing how miRNAs work. These molecules are particularly crucial for regulating genes, spreading cancer, and making treatments less effective (Li et al., 2022; Rismanchi et al., 2025). Curcumin has been shown to reduce the activity of oncogenic lncRNAs including H19 and MALAT1, which stops tumor cells from growing and epithelial mesenchymal transition (EMT) in cancers like breast, liver, and colorectal cancer. Conversely, it enhances the activity of tumor-suppressive lncRNAs such as MEG3, hence strengthening p53-mediated apoptosis and inhibiting metastasis (Cai et al., 2021). The study conducted by Wang W.-H. et al. (2018) curcumin significantly reduced the survival and proliferation of A549 lung cancer cells in a dose-dependent manner. Treatment with 0.6 μM curcumin decreased BrdU-positive cells and Cyclin D1 expression while promoting apoptosis. Curcumin suppressed the lncRNA UCA1, although its overexpression mitigated these effects. Furthermore, curcumin inhibited the Wnt and mTOR signaling pathways through the downregulation of UCA1, suggesting that curcumin exerts its anticancer effects in lung cancer by targeting the UCA1–Wnt/mTOR axis (Wang W.-H. et al., 2018). Another study by Zheng et al. (2021) revealed that the inhibitory effect of curcumin on the growth of cisplatin-resistant colorectal cancer cells was diminished by the ectopic expression of KCNQ1OT1. KCNQ1OT1 intensified cisplatin resistance in colorectal cancer cells via the miR-497/Bcl-2 pathway. Curcumin treatment effectively downregulates KCNQ1OT1 expression, hence restoring cisplatin resistance in colorectal cancer cells (Zheng et al., 2021). circRNAs are generated through the back splicing of precursor mRNAs and serve multiple roles, including acting as miRNA sponges. Research has demonstrated that curcumin modulates several circRNAs, including circ-HN1, circ-PRKCA, circPLEKHM3, circZNF83, circFNDC3B, circ_KIAA1199, circRUNX1, circ_0078710, and circ_0056618. The modification of these circRNAs influenced mRNA expression and altered different signaling cascades and cancer markers (Si et al., 2023).

### 3.4 RNA methylation and curcumin

#### 3.4.1 m^6^A is the most abundant internal mRNA modification

It is the METTL3/METTL14 complex, erased by demethylases such as FTO and ALKBH5, and decoded by proteins (e.g., YTHDFs, IGF2BPs). These reader proteins determine mRNA splicing, stability, localization, and translation. Curcumin interacts with this machinery at multiple points by promoting or inhibiting the activity of specific writers, erasers, or readers. For instance, curcumin can upregulate or downregulate writer and eraser expression, thereby altering m^6^A levels on target mRNAs. Additionally, by affecting the binding of proteins to readers, curcumin can change the fate of specific oncogenic or stress-related transcripts. This reshapes translational output and ultimately positions curcumin as an epitranscriptomic modulator that restructures post-transcriptional gene expression in cancer and inflammation (Song et al., 2022).

#### 3.4.2 Cancer-focused evidence

In prostate cancer models (PC3, DU145), curcumin reduces the stability of the oncogenic m^6^A-modified circRNA (circ0030568) by suppressing METTL3 writer dependent methylation, which adds m^6^A marks, and down-regulating the YTHDF2 that normally binds and protects circ0030568 from degradation. Destabilizing circ0030568, in turn, inhibits proliferation and migration. *In vitro*, curcumin was effective in exposures ranging from 1–50 μM, with prominent effects at ≈10 µM. Mechanistic mapping further revealed that curcumin’s m^6^A remodeling acts through a circ0030568-FMR1 axis to modulate RNA stability. These data provide a direct link between curcumin, m^6^A regulators, and anticancer activity via RNA stability (Sun et al., 2024).

#### 3.4.3 Inflammation and metabolic contexts

Beyond cancer, curcumin increased m^6^A on TRAF4 mRNA by inhibiting ALKBH5, which in turn enhanced YTHDF1-dependent TRAF4 translation and suppressed adipogenesis. This pinpoints an anti-obesity mechanism via epitranscriptomic control. Similarly, nano-engineered curcumin (curcumin-carbon dots) downregulated METTL3 and reduced m^6^A on IRE1α, thereby blunting inflammation and promoting bone healing in periodontitis models (*in vitro*/*in vivo*), which highlights tissue-repair applications. Finally, emerging neuroinflammation work suggests curcumin can influence YTHDF2 signaling/phosphorylation, implicated in its microglial-modulating effects. Together, these studies underscore a consistent theme: curcumin rebalances writer/eraser/reader activities to retune mRNA stability and translation in disease-relevant pathways (Chen Y. et al., 2021) Table 5.

**TABLE 5:** Experimental Evidence of Curcumin and m^6^A Modulation.

Model (*in vitro*/*in vivo*)	Context/Disease	Curcumin format/derivative and dose	m6A Regulator(s) affected	Target RNA(s)	Key findings/Phenotype	References
*In vivo* (piglets) + intestinal tissue	Weaning piglets, gut barrier	Dietary curcumin + resveratrol (300 mg/kg each) for 28 days	↓ global m6A; effects on m6A enrichment of transcripts	OCLN, CLDN1, ZO-1, HO-1 transcripts	Reduced m^6^A enrichment, stabilized tight junction and antioxidant mRNAs, improved mucosal integrity	Gan et al. (2019)
*In vivo* (piglets)	Liver injury/metabolic inflammation	Dietary curcumin (in LPS-challenged piglets)	Altered METTL3, METTL14, ALKBH5, FTO, YTHDF2 mRNA; ↑ total m6A	Various hepatic mRNAs (indirect)	Attenuated lipid dysregulation, liver injury	Lu et al. (2018)
*In vitro* (osteogenic cells) + *in vivo* (periodontitis)	Inflammation/bone healing	CUR-CD (dose per paper)	↓ METTL3	IRE1α mRNA	Reduced m^6^A on IRE1α, lowered IRE1α expression, decreased inflammation, enhanced bone repair	Li et al. (2025)
*In vitro* (cancer cell lines)	Prostate cancer	Curcumin (µM range)	↓ YTHDF2 (reader) and modulation of METTL3	circ0030568 (m6A-modified circRNA)	Destabilization of circ0030568, reduced proliferation and migration	Sun et al. (2024)
*In vitro*/*in vivo* microglial models	Neuroinflammation	Curcumin (dose per study)	Modulation of reader signaling (YTHDF2)	Inflammatory mRNAs (e.g. PTPRZ1/related)	Altered m^6^A modifications linked to microglial response	Zhang et al. (2025)

Abbreviations: m^6^A, N^6^-methyladenosine; METTL3/14, methyltransferase-like 3/14 (m^6^A “writers”); WTAP, Wilms’ tumor 1–associating protein; FTO, fat mass and obesity-associated protein (m^6^A “eraser”); ALKBH5, alkB homolog 5 (m^6^A “eraser”); YTHDF1/2, YTH, domain family protein 1/2 (m^6^A “readers”); IGF2BP, insulin-like growth factor 2 mRNA-binding protein (m^6^A “reader”); OCLN, occludin; CLDN1, claudin-1; ZO-1, zonula occludens-1; HO-1, heme oxygenase-1; IRE1α, inositol-requiring enzyme 1 alpha; TRAF4, TNF, receptor–associated factor 4; circ0030568, circular RNA, 0030568; PTPRZ1, protein tyrosine phosphatase receptor type Z1; CUR-CD, curcumin-carbon dots; LPS, lipopolysaccharide; ↑, increased; ↓, decreased; *in vitro*, performed in cultured cells*; in vivo*, performed in living organisms.

### 3.5 Summary of curcumin’s epigenetic effects

**FIGURE 7:**
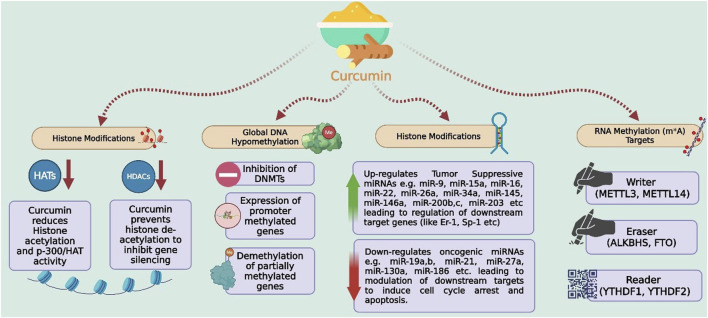
Epigenetic regulation of gene expression by curcumin. Curcumin modulates multiple epigenetic targets, including inhibition of DNA methyltransferases (DNMTs), suppression of histone acetyltransferases (HATs), and histone deacetylases (HDACs), leading to global DNA hypomethylation and reactivation of silenced tumor suppressor genes. It also regulates microRNAs (miRNAs) by upregulating tumor-suppressive and downregulating oncogenic species, and influences RNA methylation (m^6^A) machinery through writers (METTL3, METTL14), erasers (ALKBH5, FTO), and readers (YTHDF1, YTHDF2), contributing to cell cycle arrest and apoptosis. Abbreviations: DNMTs, DNA methyltransferases; HATs, histone acetyltransferases; HDACs, histone deacetylases; miRNAs, microRNAs; m^6^A, N^6^-methyladenosine.

Curcumin acts as a versatile epigenetic modulator influencing multiple layers of gene regulation Figure 7 and Table 6. It exerts DNA hypomethylating effects by inhibiting DNMTs (DNMT1, DNMT3A, DNMT3B), thereby reactivating silenced tumor-suppressor genes such as p16, RASSF1A, and SFRP1 (Hassan et al., 2019). Through histone modification, curcumin downregulates histone acetyltransferases (p300/CBP) and histone deacetylases (HDAC1, HDAC3), restoring normal chromatin architecture and transcriptional activity of growth-regulating genes (Reuter et al., 2011). It also modulates non-coding RNAs, up-regulating tumor-suppressive miRNAs (e.g., miR-34a, miR-200c) and down-regulating oncogenic ones (e.g., miR-21, miR-19a), resulting in decreased proliferation and enhanced apoptosis (Boyanapalli and Kong, 2015). Recently, curcumin has been shown to affect RNA methylation (m^6^A) by regulating writer (METTL3/METTL14), eraser (ALKBH5/FTO), and reader (YTHDF1/2) proteins, thereby altering mRNA stability and translation in cancer, inflammatory, and metabolic contexts (Sun et al., 2024; Li et al., 2025).

**TABLE 6:** Comparative summary of Curcumin’s modulation of epigenetic mechanisms across different cancer models.

Epigenetic mechanism	Molecular targets/Regulators	Cancer type/Model	Experimental evidence (*in vitro*/*in vivo*)	Effect of curcumin	References
DNA Methylation	DNMT1, DNMT3A, DNMT3B; ↑ RASSF1A, p16, SFRP1	Colon, Breast, Lung, Prostate Cancers	*In vitro* (cell lines: MCF-7, HT-29, A549); *in vivo* (xenografts)	Inhibits DNMTs → demethylation of tumor-suppressor gene promoters → apoptosis and growth inhibition	Hassan et al. (2019)
Histone Modification	HATs (p300/CBP), HDAC1, HDAC3	Leukemia, Breast Cancer, Colorectal Cancer	*In vitro* (HeLa, SW480); *in vivo* (mouse models)	Inhibits HDACs and HATs → ↑ histone acetylation at p21, p53 promoters → cell-cycle arrest	Reuter et al. (2011)
Non-coding RNAs	miR-21 ↓, miR-34a ↑, miR-200c ↑, lncRNA HOTAIR ↓	Breast, Lung, Pancreatic Cancers	*In vitro* (cell lines A549, MDA-MB-231); *in vivo* (xenografts)	Reprograms oncogenic miRNA/lncRNA network → suppressed EMT, migration and angiogenesis	Boyanapalli and Kong (2015)
RNA Methylation (m6A)	METTL3 ↓, ALKBH5 ↑, YTHDF2 ↓; targets: circ0030568, IRE1α, TRAF4	Prostate Cancer, Periodontitis, Obesity, Liver Inflammation	*In vitro* (cell lines PC3, DU145, osteogenic cells); *in vivo* (piglets, mice)	Modulates m^6^A writers/erasers/readers → ↓ oncogenic transcripts stability, ↓ inflammation, ↑ bone healing	Li et al. (2025)

Abbreviation: DNMTs, DNA methyltransferases; HATs, histone acetyltransferases; HDACs, histone deacetylases; miR, microRNA; lncRNA, long non-coding RNA; m^6^A, N^6^-methyladenosine; EMT, epithelial-mesenchymal transition; ↑, increased; ↓, decreased; *in vitro*, cell-based experiments; *in vivo*, animal models

## 4 Safety profile and toxicity concerns of curcumin

Curcumin is acclaimed for its extensive medicinal qualities, encompassing anticancer, anti-inflammatory, and antioxidant activities. Although it is extensively utilized in both traditional and contemporary medicine, comprehending its safety profile and toxicity issues is essential for its therapeutic use.

### 4.1 General safety profile: historical and clinical perspective

Curcumin has been utilized as a culinary spice for generations, with no notable side effects documented in traditional contexts. The historical application in Ayurvedic and Chinese medicine corroborates its safety when ingested in its natural state. The FDA designates curcumin as Generally Recognized As Safe (GRAS) for use as a food additive. Clinical studies validate the safety of low curcumin concentrate to moderate dosages. Lao et al. (2006) determined that oral administration of curcumin at levels of up to 8 g per day for 3 months was well-tolerated by people. Nonetheless, individual variability and formulation discrepancies may affect safety profiles. Short-term studies frequently indicate minor side effects, including nausea and gastrointestinal distress (Cheng et al., 2001; Sharma et al., 2004; Lao et al., 2006).

### 4.2 Potential adverse effects at high doses

Although curcumin is often safe at research-based dosages, excessive amounts may result in negative effects. Research indicates that elevated doses may cause gastrointestinal issues, such as nausea, diarrhoea, and abdominal discomfort (Dulbecco and Savarino, 2013). A study showed moderate gastrointestinal effects in persons ingesting curcumin levels beyond 6 g per day. Preclinical studies have demonstrated hepatotoxicity at elevated doses. In rodents, acute toxicity studies indicate that curcumin dosages exceeding 2000 mg/kg may modify liver enzymes, implying possible hepatic stress (Sirasanagandla et al., 2022). Moreover, elevated dosages may disrupt iron metabolism, perhaps resulting in iron deficiency anaemia (Tiekou Lorinczova et al., 2020).

#### 4.2.1 Pro-oxidant effect of curcumin at higher doses

Curcumin is often heralded for its antioxidant properties. It scavenges ROS and protects normal cells from oxidative damage. Many studies emphasize its capacity to upregulate endogenous antioxidant defenses and suppress oxidative stress in inflammatory and precancerous settings (Jakubczyk et al., 2020). However, at higher concentrations or in the presence of transition metal ions (e.g. Cu^2+^, Fe^2+^/Fe^3+^), curcumin can paradoxically act as a pro-oxidant. It catalyzes ROS generation that overwhelms cancer cell redox homeostasis (Jung et al., 2016). This pro-oxidant effect is especially relevant in malignant cells, which often already exist under elevated basal oxidative stress. Tipping the balance further can trigger oxidative damage and mitochondrial dysfunction selectively in tumor cells.

In cancer, this pro-oxidant spike induced by curcumin can serve as a lethal switch. Excess ROS damages mitochondrial membranes, oxidizes nucleic acids, lipids, and proteins, and activates apoptotic cascades via cytochrome c release and caspase activation (Hu et al., 2024). While its antioxidant activity may protect normal cells or basal tissues, its pro-oxidant activity at supra-physiological doses or in metal-rich microenvironments becomes a mechanism for curcumin to selectively kill cancer cells. Incorporating this dual behavior into your cancer section will strengthen mechanistic insight. The pro-oxidant action often underpins curcumin’s apoptosis-inducing and anticancer effects.

#### 4.2.2 Gastrointestinal and hypertensive side effects of curcumin at high doses

Although curcumin is best known for its anti-inflammatory and antioxidant properties, at high concentrations it can influence COX enzymes in a way that may lead to gastrointestinal or vascular side effects. Unlike selective COX-2 inhibitors (coxibs), which block COX-2 activity while sparing COX-1, curcumin acts as a partial and condition-dependent inhibitor of both COX-1 and COX-2. At lower or therapeutic concentrations, curcumin predominantly downregulates COX-2 and reduces prostaglandin E_2_ (PGE_2_) synthesis. It does this by modulating NF-κB and AP-1 pathways, thereby exerting anti-inflammatory and antitumor effects with minimal gastrointestinal irritation (Goel et al., 2001). However, at higher doses or in the presence of oxidative stress and transition metals, curcumin can also interfere with COX-1 activity. This may potentially compromise mucosal protection and theoretically increase the risk of gastric irritation or ulceration.

This partial inhibition profile distinguishes curcumin from both non-selective NSAIDs and selective COX-2 inhibitors. Non-selective COX inhibitors, such as ibuprofen and indomethacin, can induce gastric ulcers and hypertension by suppressing COX-1 mediated prostaglandin synthesis. Selective COX-2 inhibitors reduce gastrointestinal damage but may predispose patients to myocardial infarction and thrombosis by decreasing prostacyclin levels. In contrast, curcumin demonstrates preferential inhibition of COX-2 over COX-1. This allows anti-inflammatory efficacy while maintaining a favorable safety margin. Therefore, at pharmacological or dietary doses, curcumin is generally considered safe. However, at supraphysiological doses, its partial COX inhibition may contribute to mild gastrointestinal or hypertensive effects, emphasizing the need for balanced dosing and further clinical evaluation (Mohan et al., 2021).

### 4.3 Long-term toxicity concerns

The prolonged use of curcumin, particularly at elevated dosages, prompts concerns over its possible toxicological effects. No significant adverse consequences have been documented in long-term human research; nevertheless, animal models have indicated reproductive toxicity and development retardation at excessively high doses (Gota et al., 2010). Moreover, curcumin’s capacity to inhibit cytochrome P450 enzymes may influence the metabolism of numerous medicines over extended durations. Oxidative damage constitutes an additional concern. Despite curcumin’s status as an antioxidant, excessive use may, paradoxically, exacerbate oxidative stress under specific circumstances. Extended investigations in people are essential to delineate a more comprehensive understanding of its safety profile (Rodríguez Castaño et al., 2019).

### 4.4 Drug interactions

The interaction of curcumin with other pharmaceuticals is a notable concern, especially for people receiving chemotherapy or anticoagulants. Curcumin’s capacity to influence cytochrome P450 enzymes may alter the metabolism of chemotherapeutic drugs like paclitaxel and doxorubicin, potentially augmenting or diminishing their effectiveness (Anand et al., 2007). Moreover, curcumin’s anti-inflammatory characteristics may interact with NSAIDs, improving therapeutic results while also heightening the risk of side effects. Curcumin possesses anticoagulant characteristics, potentially interacting with medications such as warfarin or aspirin, hence elevating the risk of hemorrhagic consequences. Meticulous oversight and dosage modifications are required when curcumin is concurrently provided with other therapies (Peng et al., 2021).

### 4.5 Formulation strategies to enhance efficacy and safety

**FIGURE 8:**
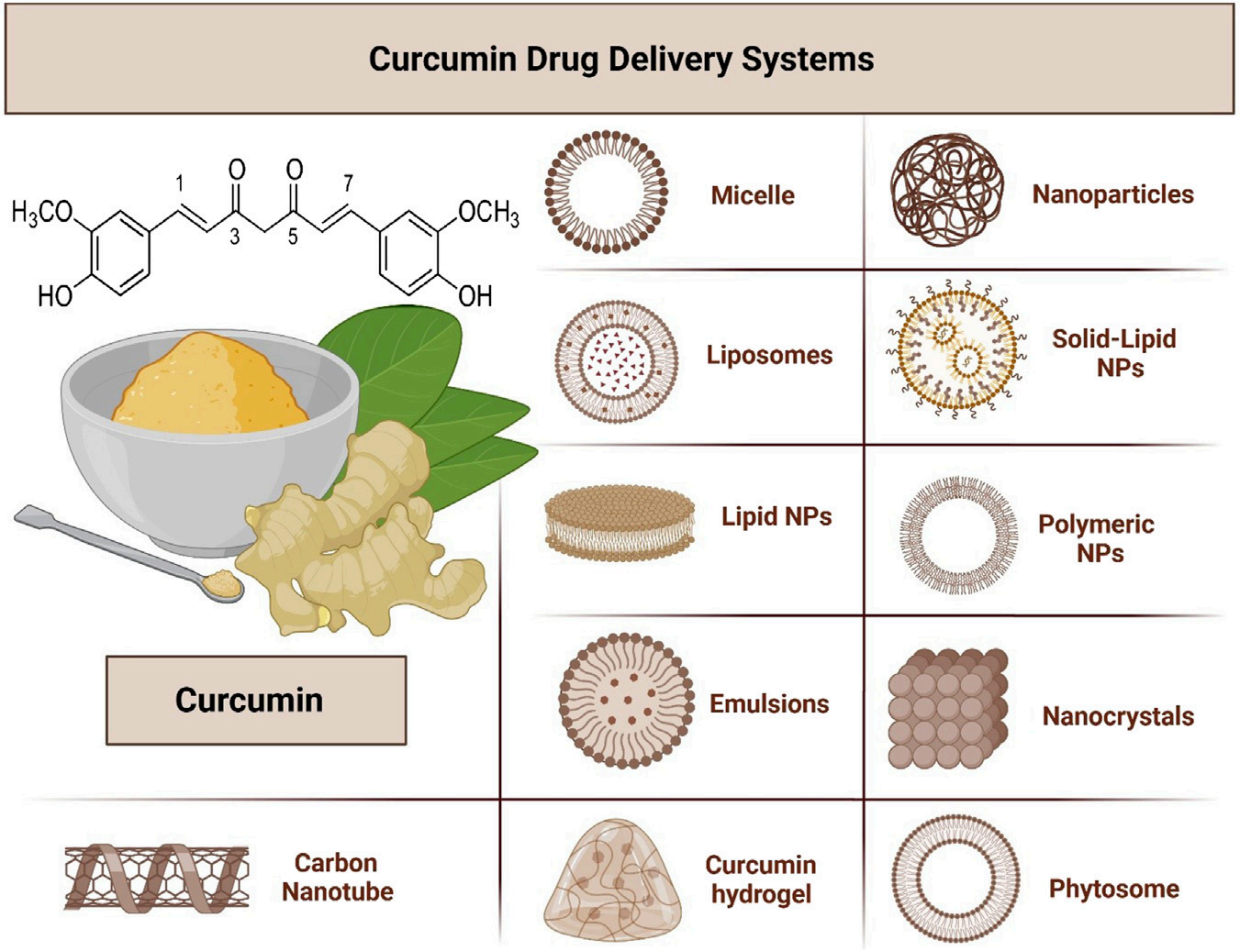
The various advanced drug delivery systems designed to enhance the bioavailability and therapeutic efficacy of curcumin. Curcumin, a hydrophobic compound derived from *Curcuma longa*, faces challenges in clinical applications due to its poor solubility, stability, and bioavailability. To overcome these limitations, multiple drug delivery platforms have been developed. The delivery systems illustrated include micelles, nanoparticles, and solid-lipid nanoparticles (NPs), which improve curcumin’s solubility and stability. Liposomes and lipid-based nanoparticles (lipid NPs) enhance the encapsulation and controlled release of curcumin. Polymeric nanoparticles (NPs) offer targeted delivery capabilities, while nanocrystals increase curcumin’s dissolution rate. Emulsions serve as simple and effective carriers for curcumin’s solubilization. Additionally, innovative systems such as phytosomes, curcumin hydrogels, and carbon nanotubes are highlighted. Phytosomes are complexes that improve curcumin’s absorption by interacting with phospholipids, while curcumin hydrogels provide sustained release and localized delivery. Carbon nanotubes, with their unique structural properties, enable efficient drug loading and transport.

The limited bioavailability of curcumin has prompted the creation of sophisticated formulation strategies to improve its efficacy and safety. Its clinical application is constrained by inadequate water solubility, diminished bioavailability, and fast metabolism. Innovative drug delivery technologies have been created to address these issues, such as liposomes, nanoparticles, and hydrogels (Rahimi et al., 2016) (Figure 8).

#### 4.5.1 Nanoparticle-based delivery systems

Nanoparticles, often measuring between 1 and 100 nm, offer an enhanced delivery mechanism for curcumin. Nanoparticles, such as polymeric nanoparticles, metal-based nanoparticles, lipid-based nanoparticles, and nanostructured lipid carriers (NLCs), are extensively utilized to enhance the bioavailability and therapeutic efficiency of curcumin. These methods encapsulate curcumin, safeguarding it from metabolic breakdown and enhancing its solubility.

Polymeric nanoparticles, including those composed of poly lactic-co-glycolic acid (PLGA), have been thoroughly investigated for the delivery of curcumin (Gracia et al., 2018). Kumari et al. (2021) conducted a study revealing that PLGA nanoparticles encapsulating curcumin markedly improved its anticancer efficacy while diminishing systemic toxicity. Roshan et al. (2024) conducted a study indicating that PLGA-curcumin nanoparticles enhanced the half-life of curcumin and augmented its anticancer effectiveness in a murine model of breast cancer. Lipid-based nanoparticles, such as solid lipid nanoparticles (SLNs) and NLCs, are highly efficient in encapsulating hydrophobic chemicals, including curcumin. Sadegh Malvajerd et al. (2019) found that curcumin-loaded solid lipid nanoparticles improved cerebral delivery and exhibited promise in the treatment of neurodegenerative disorders (Figure 8).

Metallic nanoparticles, including gold and silver nanoparticles, have been studied for transporting curcumin. These nanoparticles can augment curcumin’s therapeutic capabilities via their inherent photothermal and antibacterial activity. Curcumin-gold nanoparticles exhibit potential in the application of photothermal therapy for cancer treatment (Akhtar et al., 2022).

#### 4.5.2 Liposomal encapsulation

Liposomal versions of curcumin encapsulate the molecule in a phospholipid bilayer, enhancing its solubility and cellular absorption. Liposomes are spherical vesicles consisting of one or more phospholipid bilayers capable of encapsulating both hydrophilic and hydrophobic substances. Their biocompatibility, capacity to safeguard curcumin against degradation, and improved cellular absorption render them a suitable platform for curcumin delivery (Figure 8) (Dejeu et al., 2024). Liposomal curcumin has demonstrated the potential to mitigate gastrointestinal toxicity and improve anticancer activity in preclinical studies (Farghadani and Naidu, 2022). The incorporation of targeted ligands can augment the specificity and safety of liposomal curcumin.

Research indicates that curcumin-encapsulated liposomes markedly improve its bioavailability and therapeutic effectiveness. A study by (Laurindo et al., 2023) revealed that curcumin liposomes enhanced medication retention duration and augmented anti-inflammatory efficacy in an animal model of colitis (Zhang et al., 2023). similarly showed that liposomal curcumin demonstrated enhanced antitumor efficacy both *in vitro* and *in vivo* relative to free curcumin. Liposomes may be functionalized with targeted ligands, including folic acid or antibodies, to enhance selectivity for pathological tissues.

#### 4.5.3 Cyclodextrin complexes

Cyclodextrins are cyclic oligosaccharides that form inclusion complexes with hydrophobic compounds like curcumin, enhancing its water solubility (Figure 8). In animal studies, these complexes demonstrated improved absorption and reduced gastrointestinal side effects (Yadav et al., 2009). The complexation of curcumin with cyclodextrin diminishes the potential for interactions with other medications, hence improving its safety profile.

#### 4.5.4 Combination therapies

Combining curcumin with other medications can enhance its therapeutic effectiveness and mitigate any adverse effects. For example, piperine combined with curcumin, significantly slows its metabolic breakdown and enhances bioavailability by up to 2000% (Shoba et al., 1998). Furthermore, curcumin combined with resveratrol or quercetin has exhibited synergistic effects in reducing oxidative stress and inflammation.

#### 4.5.5 Sustained-release formulations

Sustained-release technologies, such as hydrogels and microspheres, provide a regulated release of curcumin, reducing dose-dependent toxicity and sustaining therapeutic levels over prolonged durations. A study by Jose et al. (2024) crevealed that pH-sensitive hydrogels containing curcumin mitigated gastrointestinal adverse effects and improved wound healing in diabetic rats. Hydrogels are three-dimensional, cross-linked polymer networks that may retain significant quantities of water or biological fluids. They provide a multifaceted platform for prolonged and localized drug delivery, rendering them appropriate for curcumin administration (Sood et al., 2023). Curcumin-encapsulated hydrogels have demonstrated efficacy in promoting wound healing, mitigating inflammation, and facilitating tissue regeneration (Kamar et al., 2019), conducted a study revealing that a hydrogel infused with curcumin markedly expedited wound repair in a diabetic rat model. Additionally, hydrogels can be designed for stimuli-responsive drug release, including pH or temperature-sensitive variants, to deliver curcumin under specified physiological conditions (Mahdian et al., 2023).

#### 4.5.6 Biosafety and toxicity assessment of curcumin and its nanoformulations

Curcumin, as a pure compound, is generally considered safe in humans at moderate to high oral doses. Its nanoformulations, however, may introduce new safety considerations. Clinical trials have safely administered oral curcumin up to 8 g/day. Only mild gastrointestinal side effects, such as diarrhea, are reported at this upper range (Sharma et al., 2004). In animal and cell models, curcumin shows low cytotoxicity toward normal cells. Dose-dependent effects may emerge when concentrations exceed certain thresholds or when exposure is prolonged.

Nanoformulations of curcumin, such as nanoparticles, liposomes, and micelles, enhance tissue delivery and cellular uptake. These forms may also alter toxicity profiles. *In vivo* studies of curcumin nanoparticles (Cur-NP) across breast cancer models reported no significant hematologic or organ toxicity. They also found no weight loss and normal biochemical markers compared to controls (Ombredane et al., 2021). Conversely, alveolar macrophage models exposed to curcumin nanoparticles have shown toxicity. This toxicity correlates with surface polarity changes. This finding indicates that nanoparticle surface chemistry is critical for safety (Loo et al., 2022). Moreover, a review of curcumin-based nanoformulations summarized that most designs employ biocompatible polymers and coatings to mitigate toxicity. Nevertheless, certain formulations evoked oxidative stress or mitochondrial perturbations at high doses (Hafez Ghoran et al., 2022).

Regulatory frameworks classify curcumin as GRAS for intended food additive and flavoring uses. This classification is based on historical data and toxicology (GRAS, 2018). However, its use as a therapeutic agent or in high-dose nanoformulations requires formal safety validation per drug regulatory guidelines. These include GLP toxicology, genotoxicity, and immunotoxicity assays. A cautionary case is the FDA’s (2017) investigation of a compounded curcumin emulsion product containing non-pharmaceutical grade PEG-40 castor oil. This product was implicated in hypersensitivity, contamination (diethylene glycol), and fatal adverse events (FDA, 2017). Thus, while curcumin has a favorable safety record, nanoformulations must be judiciously assessed for off-target effects, dose ceilings, and carrier-associated toxicity.

##### 4.5.6.1 Nanotoxicology and biocompatibility of curcumin-based nanocarriers

Recent advancements in nanotechnology-driven curcumin delivery systems show a shift from conventional carriers to advanced hybrid nanoplatforms. These platforms aim to optimize targeted delivery, control drug release, and improve therapeutic specificity. Stimuli-responsive nanoparticles release curcumin in response to pH, temperature, redox potential, or enzymatic activity changes. This response enhances tumor penetration and reduces systemic toxicity, making them promising tools for targeted cancer therapy (Kenchegowda et al., 2021; Qiu et al., 2021) Hybrid nanosystems combine organic components (like liposomes and polymers) with inorganic elements (silica, gold, or magnetic nanoparticles). This combination merges biocompatibility with imaging or hyperthermic functionalities. The result is valuable theranostic applications (Seaberg et al., 2021). Recently, bioengineered nanozymes and biomimetic nanocarriers, such as enzyme-mimicking nanoparticles or exosomes, have gained attention. These platforms autonomously release drugs and catalyze reactions in response to environmental changes. This feature enhances the availability and efficacy of curcumin as a therapeutic agent (Sun et al., 2012; Iroegbu et al., 2025). Bioengineered nanozymes provide catalytic functions for curcumin carriers, such as ROS-scavenging or pro-oxidant activity in tumor microenvironment. Carbon-based curcumin dots are used for antimicrobial, wound-healing, or precision periodontal therapy. Fe–curcumin nanozymes and MOF-nanozyme platforms offer both diagnostic and therapeutic catalytic augmentation. Curcumin carbon-dot systems, including Zn-doped and alendronate-targeted versions, show tissue targeting and microenvironment modulation. Notably, they reveal emerging epitranscriptomic links (e.g., IRE1α axis) in inflammatory bone disease (Yuan et al., 2022).

Stimuli-responsive nanocarrier systems exploit both internal (pH, redox potential, and enzymatic activity) and external (light, magnetic, or thermal) triggers to achieve on-target drug release while minimizing systemic exposure. In oncology and inflammatory disorders, these smart designs enable curcumin delivery to be spatially and temporally controlled, improving local bioavailability and therapeutic outcomes. For instance, dual pH- and redox-responsive polymeric and micellar carriers have been optimized for curcumin, exhibiting controlled degradation and sustained intracellular release in tumor microenvironments (Pillarisetti et al., 2017). Moreover, recent frameworks extend these strategies to inflammatory and pulmonary diseases, where similar trigger-based principles guide the rational selection of linkers and cross-linking chemistries for in vitro–in vivo correlation (Kalkal et al., 2025; Vyas et al., 2025).

Recent studies further emphasize that the biosafety and efficacy of curcumin nanoformulations are governed by the physicochemical properties of their carrier matrices—particularly particle size, surface charge, and solubility (Pillarisetti et al., 2017; Singh et al., 2024). Alginate-based or metal-ion-cross-linked (Ca^2+^/Zn^2+^) composites display excellent hemostatic and hemocompatible performance with minimal cytotoxicity, supporting their use in wound-healing and anticancer therapies (Chauhan et al., 2025). Similarly, carbon-based nanostructures, including graphene oxide, carbon quantum dots, and nanodiamond hybrids—demonstrate low toxicity, strong antioxidant activity, and high drug-loading capacity, making them versatile platforms for regenerative and antimicrobial applications (Yadav S. K. et al., 2025). The development of biomimetic and electroactive hybrid systems further expands the biomedical scope of stimuli-responsive nanomaterials. For example, a two-dimensional MXene–DNA hybrid hydrogel has been engineered for highly sensitive thrombin detection, exhibiting excellent linearity and tunable resistance changes upon ligand binding, thus demonstrating the convergence of bioelectronics and nanomedicine (Morya et al., 2025). Similarly, the design of a MoS_2_@rGO@MWCNTs ternary nanohybrid electrode illustrates how multi-component, redox-active interfaces can translate into ultrasensitive electrochemical biosensors with nanometre-scale control of charge transfer and band-bending phenomena (Yadav A. K. et al., 2025). The same structural logic, layered heterojunctions, high surface conductivity, and Fermi-level modulation can be leveraged in curcumin nanoformulations to enhance drug–cell interactions, enable redox-triggered release, and support combined diagnostic–therapeutic (theranostic) functions.

### 4.6 Synergistic formulations with other therapeutic agents

Integrating curcumin with additional medicinal drugs can augment its effectiveness and expand its therapeutic uses. Synergistic formulations seek to leverage complementary modes of action, diminish medication resistance, and mitigate side effects. For example, curcumin has been integrated with standard chemotherapy agents, including doxorubicin, cisplatin, and paclitaxel, to enhance cancer treatment efficacy (Tan and Norhaizan, 2019). Ashrafizadeh et al. (2020) the co-delivery of curcumin and doxorubicin via nanoparticles improved anticancer effectiveness and decreased cardiotoxicity. Likewise, combinations of curcumin and cisplatin have shown the ability to surmount drug resistance in ovarian and lung malignancies (Afshari et al., 2023).

In addition to drug combinations, the synergistic potential of curcumin with other natural substances, including resveratrol, quercetin, and piperine, has also been well investigated. Notably, piperine increases the bioavailability of curcumin by obstructing its metabolic breakdown. Research conducted by (Shoba et al., 1998) revealed that the amalgamation of curcumin and piperine enhanced curcumin’s bioavailability in humans by 2000%. Furthermore, the concurrent treatment of curcumin and resveratrol has demonstrated increased anti-inflammatory and anticancer properties (Rapti et al., 2024).

### 4.7 Implications for personalized medicine

The incorporation of curcumin into personalized medicine frameworks is a burgeoning field of interest. Personalized medicine customizes treatment approaches according to an individual’s genetic, epigenetic, and environmental influences. Pharmacogenomic research shows that genetic changes in drug-metabolizing enzymes, transporters, and targets can affect curcumin’s pharmacokinetics and pharmacodynamics. Polymorphisms in cytochrome P450 enzymes may influence curcumin’s metabolism. This can modify its therapeutic efficacy. Recognizing these genetic variations can help refine curcumin dosage and delivery mechanisms for certain patients (Rodríguez Castaño et al., 2019). Biomarker-driven strategies can improve the accuracy of curcumin treatment. Curcumin’s anti-inflammatory properties may be especially beneficial for people with heightened inflammatory indicators, such as C-reactive protein (CRP) or interleukin-6 (IL-6). Curcumin’s anticancer efficacy may increase by targeting particular molecular pathways, such as NF-κB or epidermal growth factor receptor. These pathways are often misregulated in specific tumours (Giordano and Tommonaro, 2019; Gorabi et al., 2022). Combining genomics, proteomics, metabolomics, and microbiomics can yield new insights into curcumin’s effects. This information can inform tailored therapy approaches. Microbiome analysis can identify individuals with gut dysbiosis who may benefit from curcumin’s prebiotic effects. Metabolomics can clarify curcumin’s metabolic pathways and its interactions with other drugs or dietary elements (Manickavasagam et al., 2024).

### 4.8 Emerging applications beyond oncology

While much curcumin research focuses on cancer, growing evidence supports applications in neurological, cardiovascular, metabolic, and inflammatory diseases. For example, in neurodegenerative disease models (e.g. Alzheimer’s, Parkinson’s), curcumin nanoformulations have demonstrated blood–brain barrier penetration, suppression of neuroinflammation and amyloid aggregation, and modulation of epigenetic and signaling pathways. Correspondingly, some clinical trials (e.g. NCT01383161) are testing the memory effects of curcumin in age-related cognitive impairment (Small, 2020). In arthritis and rheumatologic conditions, 29 randomized controlled trials (∼2396 participants) treated doses ranging 120–1500 mg over 4–36 weeks; curcumin and Curcuma longa extract were safe and improved inflammation and pain outcomes (Zeng et al., 2022). Similarly, cardiovascular and metabolic disease models also benefit from curcumin interventions, often via anti-inflammatory, antioxidant, and lipid-regulatory effects. A recent review highlights curcumin’s cardioprotective and anti-metabolic syndrome potential, emphasizing that nano-curcumin increases curcumin’s bioavailability and may enhance therapeutic potency (Bertoncini-Silva et al., 2024). Additionally, nano-curcumin combined with metal oxides or targeted carriers is being explored for anti-inflammatory and antimicrobial uses beyond cancer, as these nanoformulations can enable targeted delivery, though toxicity to normal tissues remains a concern (Beyene et al., 2021). These expanding applications underscore curcumin’s versatile therapeutic promise, though each new domain demands rigorous safety, efficacy, and mechanistic validation.

## 5 Clinical translation of curcumin

**FIGURE 9:**
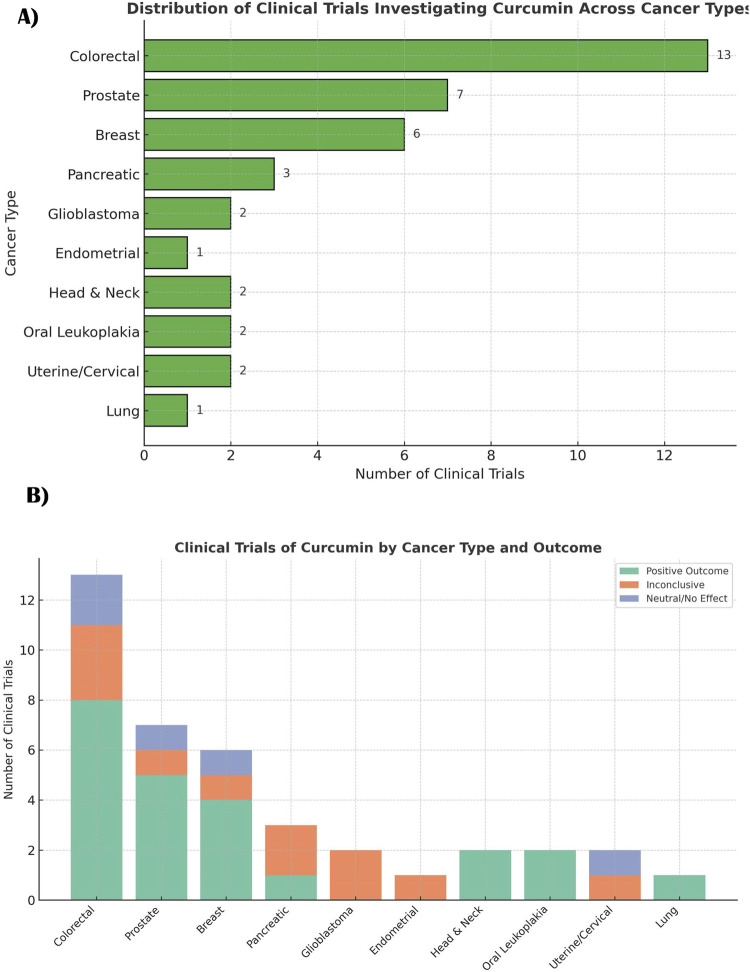
Distribution and outcome analysis of clinical trials evaluating curcumin in oncology. **(A)** shows the number of registered clinical trials per cancer type, derived from Gall Trošelj et al., Molecules, 2020, 25(22):5240 (Trošelj et al., 2020). Colorectal, prostate, and breast cancers represent the most frequently studied malignancies. **(B)** summarizes clinical outcomes across trials, illustrating that most studies demonstrated positive or adjunctive therapeutic effects, while a smaller proportion yielded inconclusive or neutral findings, often due to formulation-related bioavailability limitations.

Multiple phase I and II clinical trials have studied curcumin and its nanoformulations for several cancers, including colorectal, pancreatic, and breast, as well as chronic inflammatory illnesses. These trials show good safety and moderate efficacy (Hafez Ghoran et al., 2022). However, there are challenges in clinical use. These include low systemic exposure, differences in pharmacokinetics between formulations, and the lack of FDA/EMA standards for botanical and nanoparticle-based medicines. Also, there are no standard production methods or validated biomarkers, which makes clinical comparison harder (Yallapu et al., 2015). Table 7 lists the clinical trials on curcumin in cancer, and Figure 9 shows them.

**TABLE 7:** Summary of clinical trials investigating curcumin formulations in different types of cancer.

Cancer type	Curcumin formulation	Clinical trial reference (NCT No.)	Study type/Phase	Outcome	Key findings	References
Colorectal Cancer	C3 Complex®	NCT00113841, NCT01490996	Phase I–II	Positive	↓ Rectal ACFs (aberrant crypt foci); ↓ PGE^2^ levels; safe up to 4 g/day	Carroll et al. (2011) Garcea et al. (2004) Sharma et al. (2004)
Prostate Cancer	BCM-95®, C3 Complex®	NCT03211104, NCT01917890	Randomized, Double-Blind	Positive/Moderate	↓ PSA increase, improved oxidative status, well-tolerated	Choi et al. (2019) Hejazi et al. (2016)
Breast Cancer	CUC-1R (IV Curcumin), Meriva®	NCT03072992	Phase II	Positive	Improved overall response rate with CUC-1R + Paclitaxel	Saghatelyan et al. (2020) Ibrahim et al. (2010), Chandra et al. (2016)
Pancreatic Cancer	C3 Complex®, Meriva®, Theracurmin™	NCT02017353, NCT02017353	Phase I/II	Inconclusive	Safe up to 8 g/day PO; combination with gemcitabine feasible	Dhillon et al. (2008), Epelbaum et al. (2010), Kanai et al. (2011), Kanai et al. (2013)
Glioblastoma	NovaSol®, LongVida®	NCT01712542	Open Label	Inconclusive	Curcumin detected in tumor tissue (9–151 pg/mg); variable absorption	Dützmann et al. (2016), Gota et al. (2010)
Endometrial Cancer	Meriva®	NCT02017353	Open Label	Neutral	No significant biomarker change after 2 weeks Meriva® 2 g/day	Tuyaerts et al. (2019)
Head and Neck Cancer	C3 Complex®, BCM-95®	NCT01160302	Randomized Controlled	Positive	↓ IL-17, ↓ FGF-2; decreased mucositis severity (p < 0.001)	Latimer et al. (2015), Arun et al. (2020)
Oral Leukoplakia/Precancerous	BCM-95®, C3 Complex®	NCT00365209	Phase II	Positive	Durable clinical response (p = 0.02), ↓ oxidative stress markers	Kuriakose et al. (2016) Rai et al. (2010)
Uterine/Cervical Cancer	BCM-95®	NCT02017353	Phase II	Neutral	No improved response with radiotherapy + curcumin	Purbadi et al. (2020)
Lung Cancer	Lipocurc™, C3 Complex®	NCT00295035	Phase I	Preliminary	Tumor volume ↓ in mice; safe (300 mg/m2 IV); ongoing studies	Greil et al. (2018), Ranjan et al. (2016), Withers et al. (2018)

ACF, aberrant crypt foci; AE, adverse event; BCM-95® (BioCurcumax™), a patented formulation containing curcuminoids and turmeric essential oils designed to enhance bioavailability; C3 Complex®, a standardized extract of curcumin, demethoxycurcumin, and bis-demethoxycurcumin; CUC-1R, an intravenous (IV) curcumin formulation; Meriva®, a curcumin–phosphatidylcholine phytosome complex; NovaSol®, a micellar curcumin nanoformulation; LongVida®, a solid-lipid curcumin particle formulation; Theracurmin™, a colloidal nanoparticle-based curcumin; Lipocurc™, a liposomal intravenous curcumin preparation; PSA, prostate-specific antigen; PGE_2_, prostaglandin E_2_; FGF-2, fibroblast growth factor-2; IL-17, interleukin-17; PO, per os (oral administration); NCT, National Clinical Trial identifier. Clinical outcomes were categorized as Positive (demonstrated therapeutic or biomarker benefit), Inconclusive (limited or formulation-dependent evidence), or Neutral (no statistically significant improvement over control). All clinical data were obtained from registered trials on ClinicalTrials.gov and corresponding peer-reviewed publications.

Although curcumin has shown broad preclinical promise, its translation into approved therapeutics faces considerable hurdles. The U.S. FDA has not approved curcumin as a treatment for cancer or any other disease indication; it remains a dietary supplement, not a drug Clinical trials (Phase I/II) have primarily assessed safety, dosing, and biomarker modulation rather than definitive efficacy endpoints. For example, a Phase I study established that 3.6 g daily of curcumin is tolerable and biologically active (reduced PGE_2_ in leukocytes); mild diarrhea was the only dose-limiting toxicity (Sharma et al., 2004). In a Phase II trial in pancreatic cancer patients, oral curcumin was well tolerated and showed some biological activity in a subset of patients, despite low systemic absorption (Dhillon et al., 2008).

These clinical challenges are compounded by significant regulatory obstacles, including inconsistent bioavailability, batch variability, lack of standardization, and limited intellectual property protection for natural compounds. Some nanoformulation patents exist, but scaling up GMP-level manufacturing, ensuring batch reproducibility, stability, and regulatory documentation (Chemistry, Manufacturing, and Controls (CMC), toxicity, pharmacokinetics) remain challenges (Bagheri et al., 2022). Further, as curcumin is often classified as a supplement rather than a drug, many clinical trials do not adhere to rigorous IND/CTA frameworks, which complicates regulatory acceptance (NIH, 2025). There is a pressing need for well-controlled randomized trials using standardized curcumin or nano-curcumin formulations, and closer alignment with drug regulatory standards (e.g. FDA/EMA guidance on nanomedicines).

## 6 Critical analysis and comparative insights on curcumin’s epigenetic mechanisms

Curcumin stands out among natural epigenetic modulators. It targets multiple layers of the epigenetic machinery at once, a property not shared to the same extent by compounds such as resveratrol, epigallocatechin gallate (EGCG), or sulforaphane. Most phytochemicals influence either DNA methylation or histone acetylation independently. In contrast, curcumin has demonstrated coordinated inhibition of DNMTs (DNMT1, DNMT3A, DNMT3B), suppression of histone deacetylases (HDAC1, HDAC3), and modulation of non-coding RNAs and m^6^A RNA methylation within the same cellular context. This broad modulation enables curcumin to reactivate tumor-suppressor genes such as p16, RASSF1A, and SFRP1. At the same time, it silences oncogenic pathways. Compared with single-target modulators like genistein or quercetin, curcumin’s pleiotropic epigenetic actions are accompanied by anti-inflammatory, antioxidant, and anti-metastatic effects. This provides a unique integrative therapeutic advantage. Importantly, curcumin also sensitizes cancer cells to chemotherapy and reduces treatment-related side effects. For example, it enhances the efficacy of cisplatin and doxorubicin by mitigating oxidative stress and drug resistance mechanisms (Hassan et al., 2019).

## 7 Unresolved and controversial issues in curcumin epigenetic research, limitations and future directions

Curcumin’s promise in epigenetic research is tempered by significant experimental and interpretive challenges. Experimental reproducibility remains a concern because curcumin’s stability and purity vary widely across studies, leading to inconsistent bioavailability and pharmacodynamic outcomes. Furthermore, target specificity at the enzyme and chromatin levels is often unclear, as curcumin’s polyphenolic structure allows for broad molecular interactions that can complicate mechanistic interpretation (Hafez Ghoran et al., 2022). Many reported DNMT, HAT, and HDAC inhibitory effects of curcumin are concentration-dependent and not always reproducible across experimental systems. Differences in curcumin purity, solvent conditions, and cell-line sensitivity lead to variable demethylation or histone modification profiles, complicating direct comparisons between studies (Medina-Franco et al., 2011; Hassan et al., 2019). Moreover, the specificity of curcumin’s epigenetic actions remains uncertain; it interacts with multiple chromatin-modifying enzymes and transcriptional regulators, raising debate over whether its effects are targeted or stem from general redox and signaling modulation (Chmielewska-Kassassir and Wozniak, 2021). Additionally, *in vivo* validation and clinical reproducibility are limited. Few human studies confirm DNMT or histone changes in patient tissues, and observed gene-expression shifts often lack corresponding epigenetic readouts (Hassan et al., 2019; Tang et al., 2021). Bioavailability and pharmacokinetics pose major challenges. Native curcumin’s rapid metabolism and low systemic exposure question whether the intracellular concentrations required for epigenetic modulation are physiologically achievable. Although nanocarrier formulations (liposomes, micelles, solid-lipid nanoparticles) improve plasma levels, it remains controversial whether they recapitulate the same chromatin-targeting actions (Jacob et al., 2024). Curcumin may demethylate tumor-suppressor genes in cancer cells but also suppress inflammatory-gene promoters in immune cells, reflecting tissue-specific signaling crosstalk rather than a uniform epigenetic mechanism (Reuter et al., 2011). Translational limitations also persist: *in vitro* concentrations required for epigenetic modulation frequently exceed physiologically achievable plasma levels, raising questions about clinical relevance. To overcome these barriers, current research focuses on nano-curcumin and hybrid nanocarriers that improve systemic bioavailability and enable site-specific epigenetic targeting (Kumar et al., 2024; Singh et al., 2024).

To identify gaps and future work, the new text now explicitly flags: (i) standardization and reproducibility. This includes material grade, surface chemistry, and batch-to-batch variability for hybrids/MOFs; (ii) trigger fidelity *in vivo*. pH/redox/enzyme gradients can be heterogeneous and shallow. External stimuli face penetration limits; (iii) safety/nanotoxicology. Carrier-driven immunogenicity or organ accumulation is noted. We link to recent alginate Ca/Zn hemostatic composites and related hemostatic materials as relevant safety paradigms for polysaccharide/ion-crosslinked systems; (iv) regulatory translation. CMC, GMP scale-up, and device–drug classification still lag behind preclinical promise.

## 8 Conclusion

Curcumin embodies a powerful intersection between natural product chemistry and molecular oncology, exerting multi-target effects that extend from biochemical modulation to epigenetic reprogramming. Through coordinated regulation of DNA methylation, histone modification, non-coding RNAs, chromatin remodeling, and RNA methylation (m^6^A), curcumin restores disrupted epigenetic homeostasis and inhibits tumor initiation, progression, and metastasis. Its influence on RNA methylation by modulating enzymes such as METTL3, FTO, and YTHDF adds a novel layer to its anticancer profile, demonstrating its capacity to regulate mRNA stability, translation, and gene expression at a post-transcriptional level. Despite extensive preclinical validation, curcumin’s clinical application remains constrained by poor pharmacokinetics and low systemic retention. Emerging nanocarrier technologies and bioenhancers have significantly improved its solubility, stability, and therapeutic index, supporting its repositioning as a viable epigenetic therapeutic. Future research should focus on standardized nanoformulations, systems-level omics profiling, and controlled clinical trials to validate its epigenetic efficacy and safety. Collectively, curcumin stands as a paradigm of nutriepigenomics, bridging traditional herbal wisdom with modern precision oncology.
